# KLIF-associated cytoskeletal proteins in *Trypanosoma brucei* regulate cytokinesis by promoting cleavage furrow positioning and ingression

**DOI:** 10.1016/j.jbc.2022.101943

**Published:** 2022-04-18

**Authors:** Qing Zhou, Huiqing Hu, Ziyin Li

**Affiliations:** Department of Microbiology and Molecular Genetics, McGovern Medical School, University of Texas Health Science Center at Houston, Houston, Texas, USA

**Keywords:** *Trypanosoma brucei*, cytokinesis, cleavage furrow, orphan kinesin, cytoskeleton, BioID, biotinylation, CAP, cytoskeleton-associated protein, CIF2, cytokinesis initiation factor 2, FAZ, flagellum attachment zone, FRW1, Furrow 1, FPRC, FAZ tip-localized protein required for cytokinesis, KMD, kinesin motor domain, KLIF, kinesin localized to the ingressing furrow, NFD, new-flagellum daughter, OFD, old-flagellum daughter, TPM, tropomyosin, 1N1K, one nucleus and one kinetoplast, 1N2K, one nucleus and two kinetoplasts, 2N2K, two nuclei and two kinetoplasts

## Abstract

Cytokinesis in the early divergent protozoan *Trypanosoma brucei* occurs from the anterior cell tip of the new-flagellum daughter toward the nascent posterior end of the old-flagellum daughter of a dividing biflagellated cell. The cleavage furrow ingresses unidirectionally along the preformed cell division fold and is regulated by an orphan kinesin named kinesin localized to the ingressing furrow (KLIF) that localizes to the leading edge of the ingressing furrow. Little is known about how furrow ingression is controlled by KLIF and whether KLIF interacts with and cooperates with other cytokinesis regulatory proteins to promote furrow ingression. Here, we investigated the roles of KLIF in cleavage furrow ingression and identified a cohort of KLIF-associated cytoskeletal proteins as essential cytokinesis regulators. By genetic complementation, we demonstrated the requirement of the kinesin motor activity, but not the putative tropomyosin domain, of KLIF in promoting furrow ingression. We further showed that depletion of KLIF impaired the resolution of the nascent posterior of the old-flagellar daughter cell, thereby stalking cleavage furrow ingression at late stages of cytokinesis. Through proximity biotinylation, we identified a subset of cytoskeleton-associated proteins (CAPs) as KLIF-proximal proteins, and functional characterization of these cytoskeletal proteins revealed the essential roles of CAP46 and CAP52 in positioning the cleavage furrow and the crucial roles of CAP42 and CAP50 in promoting cleavage furrow ingression. Together, these results identified multiple cytoskeletal proteins as cytokinesis regulators and uncovered their essential and distinct roles in cytokinesis.

*Trypanosoma brucei* is a flagellated, unicellular parasitic protozoan causing sleeping sickness in humans and nagana in cattle in sub-Saharan Africa. The parasite alternates between the insect vector tsetse fly and the mammalian hosts and proliferates through binary fission in the midgut of the insect vector and the bloodstream of mammals. A trypanosome cell at the G1 phase of the cell cycle contains a single flagellum, which is nucleated from the basal body, exits the cell body through the flagellar pocket and then attached to the cell body through the flagellum attachment zone (FAZ) (1). During early S-phase of the cell cycle, the trypanosome cell assembles a new basal body and then assembles a new flagellum from this newly formed basal body. Following cell cycle progression, the newly assembled flagellum exits the flagellar pocket and extends toward the cell anterior. Meanwhile, a new FAZ that associates with the newly assembled flagellum is formed, which extends, in a direction that is opposite to that of the new flagellum, from the anterior tip of the new-flagellum daughter (NFD) cell toward the flagellar pocket area (2, 3). Cytokinesis initiation occurs from the anterior tip of the NFD cell, with the cleavage furrow ingressing, along a preformed cell division fold between the new and the old flagella, toward the nascent posterior of the old-flagellum daughter (OFD) cell, thereby dividing a biflagellated mother cell into two uniflagellated daughter cells (4).

*T. brucei* employs an actomyosin-independent mechanism for its unusual mode of cytokinesis, and the regulatory pathway controlling cytokinesis comprises evolutionarily conserved and trypanosome-specific regulatory proteins, which function at the anterior tip of the NFD cell (or the new FAZ tip) and the cleavage furrow to promote cytokinesis initiation, progression, and completion (5, 6, 7, 8, 9, 10, 11, 12, 13, 14, 15, 16, 17, 18). The evolutionarily conserved regulators include two protein kinases, the Polo-like kinase TbPLK and the Aurora B kinase TbAUK1, and the microtubule severing enzyme katanin60–katanin80 complex (5, 6, 7, 8). The trypanosome-specific regulators include a novel protein phosphatase named KPP1 (kinetoplastid-specific protein phosphatase 1), an orphan kinesin named KLIF (kinesin localized to the ingressing furrow), a putative EF-hand motif-containing protein named CIF2 (cytokinesis initiation factor 2), and a subset of α-helical motif-containing proteins named CIF1, CIF3, CIF4, FPRC (FAZ tip-localized protein required for cytokinesis) and FRW1 (Furrow 1). CIF1 appears to function as an orchestrator of cytokinesis, as it interacts with all of the identified cytokinesis regulators and recruits most of its interacting partner proteins except CIF4 and FPRC to the anterior tip of the NFD cell (12, 14, 15, 16, 17, 19). Some of the CIF1-interacting proteins, such as TbPLK, KPP1, CIF2, and CIF3, exert feedback regulation on CIF1 by maintaining CIF1 protein stability or maintaining CIF1 localization at the anterior tip of the NFD cell. CIF4 and FPRC, however, appear to act upstream of CIF1 by recruiting CIF1 to the anterior tip of the NFD cell (17). The complete order of actions among these cytokinesis regulators and their mechanistic roles in cytokinesis remain poorly understood and require further investigation.

The cytoskeleton of *T. brucei* cells is defined by a polarized array of subpellicular microtubules, which is duplicated semiconservatively during the cell division cycle (20). The plus ends of the subpellicular microtubules are positioned at the posterior end of the cell, and during the S-phase of the cell cycle, these microtubules start to elongate from their plus ends toward cell posterior, whereas the newly assembled flagellum extends in an opposite direction toward cell anterior. At late cell cycle stages, a nascent posterior is formed, at the ventral side of the cell near the posterior portion of the NFD cell, for the OFD cell, and membrane invagination occurs from the anterior end of the NFD cell toward the nascent posterior of the OFD cell, forming a so-called cell division fold, along which the cleavage furrow ingresses unidirectionally from the anterior of the NFD cell toward the nascent posterior of the OFD cell. At the final stage of cytokinesis, the posterior tip of the OFD cell is connected, *via* a thin thread of cytoplasm termed cytoplasmic bridge, to the posterior portion of the NFD cell, and cleavage of the cytoplasmic bridge separates the two daughter cells. Cytoskeleton-associated proteins (CAPs) are likely involved in cytokinesis, as knockdown of several CAPs in *T. brucei* caused cytokinesis defects (21, 22, 23, 24), although whether they are directly involved in cytokinesis was not established and, if they are, how they promote cytokinesis was not elucidated.

In this report, we investigated the role of the orphan kinesin KLIF in cleavage furrow ingression, identified KLIF-associated cytoskeletal proteins by proximity biotinylation (BioID), and characterized the role of KLIF-associated cytoskeletal proteins in controlling cytokinesis in the procyclic (insect) form of *T. brucei*. The results demonstrated the essential requirement of KLIF for the formation of the nascent posterior of the OFD cell to promote cleavage furrow ingression and cytokinesis completion and discovered distinct roles of several KLIF-associated cytoskeletal proteins in regulating cleavage furrow ingression and cleavage furrow positioning. These findings highlight the essential involvement of CAPs in controlling the positioning and the ingression of the cleavage furrow to ensure faithful cytokinesis in *T. brucei*.

## Results

### The kinesin motor activity of KLIF is required for KLIF function

**Figure 1:**
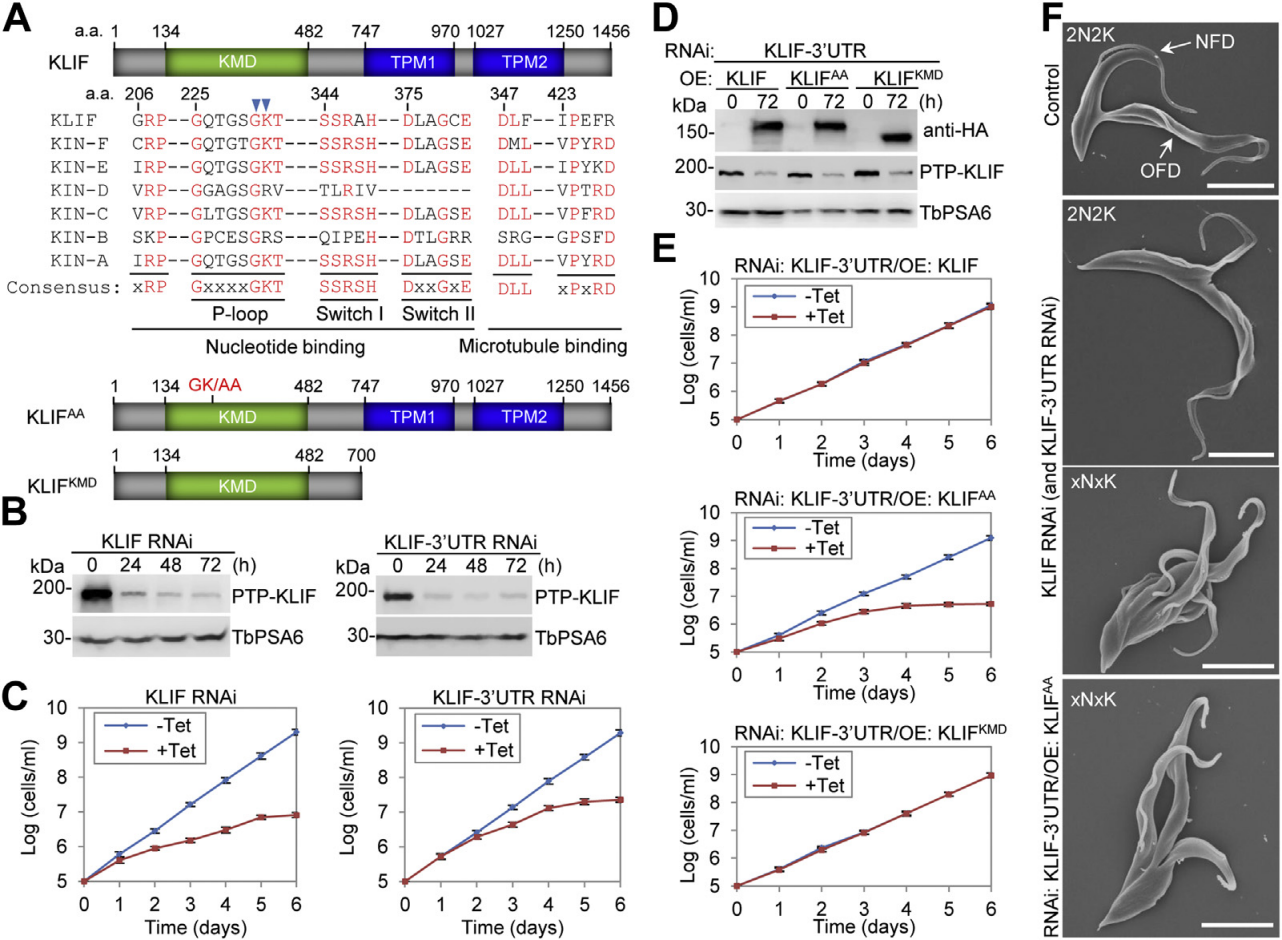
**The kinesin motor of KLIF is essential for cleavage furrow ingression**. A, schematic drawing of KLIF structural domains, sequence alignment of the conserved motifs within the kinesin motor domain (KMD), and schematic drawing of KLIF motor domain mutant and tropomyosin (TPM) domain-deletion mutant used for KLIF-3′UTR RNAi complementation. *B*, knockdown of KLIF by RNAi against the coding sequence or the 3′-UTR of KLIF in procyclic trypanosomes. KLIF was tagged with an N-terminal PTP epitope from its endogenous locus and detected by anti-Protein A antibody. TbPSA6 served as a loading control. *C*, effect of KLIF RNAi and KLIF-3′UTR RNAi on cell proliferation. *D*, genetic complementation of KLIF-3′UTR RNAi by ectopic expression of WT KLIF, KLIF motor domain mutant (KLIF^AA^), or KLIF tropomyosin domain-deletion mutant (KLIF^KMD^). Endogenous KLIF was tagged with PTP and detected by anti-Protein A antibody, whereas ectopic KLIF and its mutants were tagged with a triple HA epitope and detected by anti-HA antibody. TbPSA6 served as a loading control. *E*, growth curves of KLIF-3′UTR RNAi complementation cell lines expressing WT KLIF, KLIF^AA^, or KLIF^KMD^. *OE* in panels *D* and *E*: Overexpression. *F*, SEM images of binucleated (2N2K) and multinucleated (xNxK, x > 2) cells undergoing cytokinesis. KLIF, kinesin localized to the ingressing furrow ; NFD, new-flagellum daughter; OFD, old-flagellum daughter. Scale bars: 5 μm.

KLIF (Tb927. 8.4950) is an orphan kinesin in *T. brucei*, and it contains a putative kinesin motor domain (KMD), which consists of a nucleotide-binding motif and a microtubule-binding motif, and two putative tropomyosin (TPM) domains (Fig. 1*A*). KLIF localizes to the anterior tip of the NFD cell prior to cytokinesis initiation and to the ingressing cleavage furrow during cytokinesis and is required for cytokinesis completion in the procyclic form of *T. brucei* (12, 16). Despite its essentiality in cytokinesis, the mechanistic role of KLIF and the potential requirement of the kinesin motor activity and the TPM domains for cytokinesis have not been explored. We generated KLIF RNAi cell lines by targeting either the coding sequence or the 3′UTR of KLIF and investigated the requirement of the KMD domain and the TPM domains of KLIF for cytokinesis. To this end, we mutated the conserved glycine (G230) and lysine (K231) residues in the nucleotide-binding motif of the KMD to alanine residues to generate a KLIF^AA^ mutant and deleted the C-terminal TPM domains to generate a KLIF^KMD^ (KLIF kinesin motor domain only) mutant (Fig. 1*A*). We then tested the function of the two KLIF mutants in the KLIF-3′UTR RNAi cell line by ectopically expressing them upon induction of KLIF RNAi. RNAi of KLIF by targeting the coding sequence or the 3′UTR of KLIF both resulted in a significant reduction of KLIF protein levels, although the RNAi against the coding sequence of KLIF appeared to be more effective (Fig. 1*B*). Nonetheless, this reduction in KLIF protein levels caused severe growth defects for both RNAi cell lines (Fig. 1*C*), with the RNAi against the coding sequence of KLIF resulting in stronger growth defects than the RNAi against the 3′UTR of KLIF (Fig. 1*C*), consistent with their RNAi efficiency (Fig. 1*B*). These results demonstrated that KLIF was essential for cell proliferation in the procyclic form of *T. brucei*. In the KLIF-3′UTR RNAi complementation cell lines, ectopic expression of KLIF, KLIF^AA^, or KLIF^KMD^, which were each tagged with a C-terminal triple HA epitope, and knockdown of endogenous KLIF, which was tagged with an N-terminal PTP epitope, were confirmed by Western blotting (Fig. 1*D*). Expression of KLIF or the KLIF^KMD^ mutant restored cell proliferation of the KLIF-3′UTR RNAi cell line, whereas expression of the KLIF^AA^ mutant failed to restore cell proliferation (Fig. 1*E*), suggesting that the kinesin motor activity is essential for KLIF function and that the two putative TPM domains are not required for KLIF function.

### KLIF is required for cleavage furrow ingression in the procyclic form of *T. brucei*

We examined the effect of KLIF RNAi on the progression of the cell cycle by light microscopy and electron microscopy. Trypanosome cells at different cell cycle stage can be readily distinguished by the numbers of nucleus (N) and kinetoplast (K). A cell at stages from G1 to early S phase contains one nucleus and one kinetoplast (1N1K), a cell at stages from late S phase to early mitosis contains one nucleus and two kinetoplasts (1N2K), and a cell at stages from late mitosis to cytokinesis contains two nuclei and two kinetoplasts (2N2K). Based on this criterion, we quantitated the cells with different numbers of nucleus and kinetoplast before and after KLIF RNAi induction for both KLIF RNAi cell lines and for the KLIF-3′UTR RNAi cell line expressing the KLIF^AA^ mutant (Fig. S1*A*). The results showed that for both KLIF RNAi cell lines, the 1N1K and 1N2K cells were gradually decreased, whereas the 2N2K cells were increased at 24 h of RNAi induction and then decreased at 48 h of RNAi induction, and there was a significant increase of cells containing multiple nuclei and multiple kinetoplasts (xNxK, x > 2) to ∼65% (KLIF RNAi) and ∼54% (KLIF-3′UTR RNAi) of the total population at 48 h of RNAi (Fig. S1*A*). Similar phenotypes were also observed for the KLIF-3′UTR RNAi cell line expressing the KLIF^AA^ mutant (Fig. S1*A*). The increase of 2N2K cells initially and xNxK cells subsequently after KLIF RNAi or expression of the KLIF^AA^ mutant suggests defects in cytokinesis.

To further characterize the cytokinesis defects caused by KLIF RNAi and expression of the KLIF^AA^ mutant, we quantitated the binucleated (2N2K) cells and multinucleated (xNxK) cells that contained a visible cleavage furrow (dividing) or no cleavage furrow (nondividing) for the three cell lines. For both KLIF RNAi cell lines, there was a significant increase of the binucleated (2N2K) cells with a visible cleavage furrow from ∼31% to ∼62% (KLIF RNAi) or to ∼50% (KLIF-3′UTR RNAi) after 48 h of RNAi induction (Fig. S1*B*). Strikingly, the multinucleated (xNxK) cells with multiple visible cleavage furrows emerged to ∼88% (KLIF RNAi) or ∼56% (KLIF-3′UTR RNAi) after 48 h of RNAi induction (Fig. S1*B*). Similarly, expression of the KLIF^AA^ mutant in the KLIF-3′UTR RNAi cell line also caused a significant increase of the binucleated (2N2K) cells with a visible cleavage furrow from ∼31% to ∼55% and the emerge of multinucleated (xNxK) cells to ∼65% after 48 h of induction (Fig. S1*B*). Analysis by scanning electron microscopy (SEM) showed that the cleavage furrow in the KLIF RNAi cell lines and the KLIF-3′UTR RNAi cells expressing the KLIF^AA^ mutant failed to ingress to its end toward the nascent posterior of the OFD cell of the binucleated (2N2K) cells as well as the multiple nascent posteriors of the multinucleated (xNxK) cells (Fig. 1*F*). In contrast, in the noninduced control binucleated (2N2K) cells, the cleavage furrow was able to ingress until its end, resulting in the connection of the nascent posterior of the OFD cell to the ventral side of the NFD cell (Fig. 1*F*). This finding suggests that cleavage furrow ingression was stalled mid-way during the ingression process when KLIF was depleted or lost its kinesin motor activity. Together, these results demonstrated that KLIF and its kinesin motor activity were required for cleavage furrow ingression in the procyclic form of *T. brucei*.

**Figure 2:**
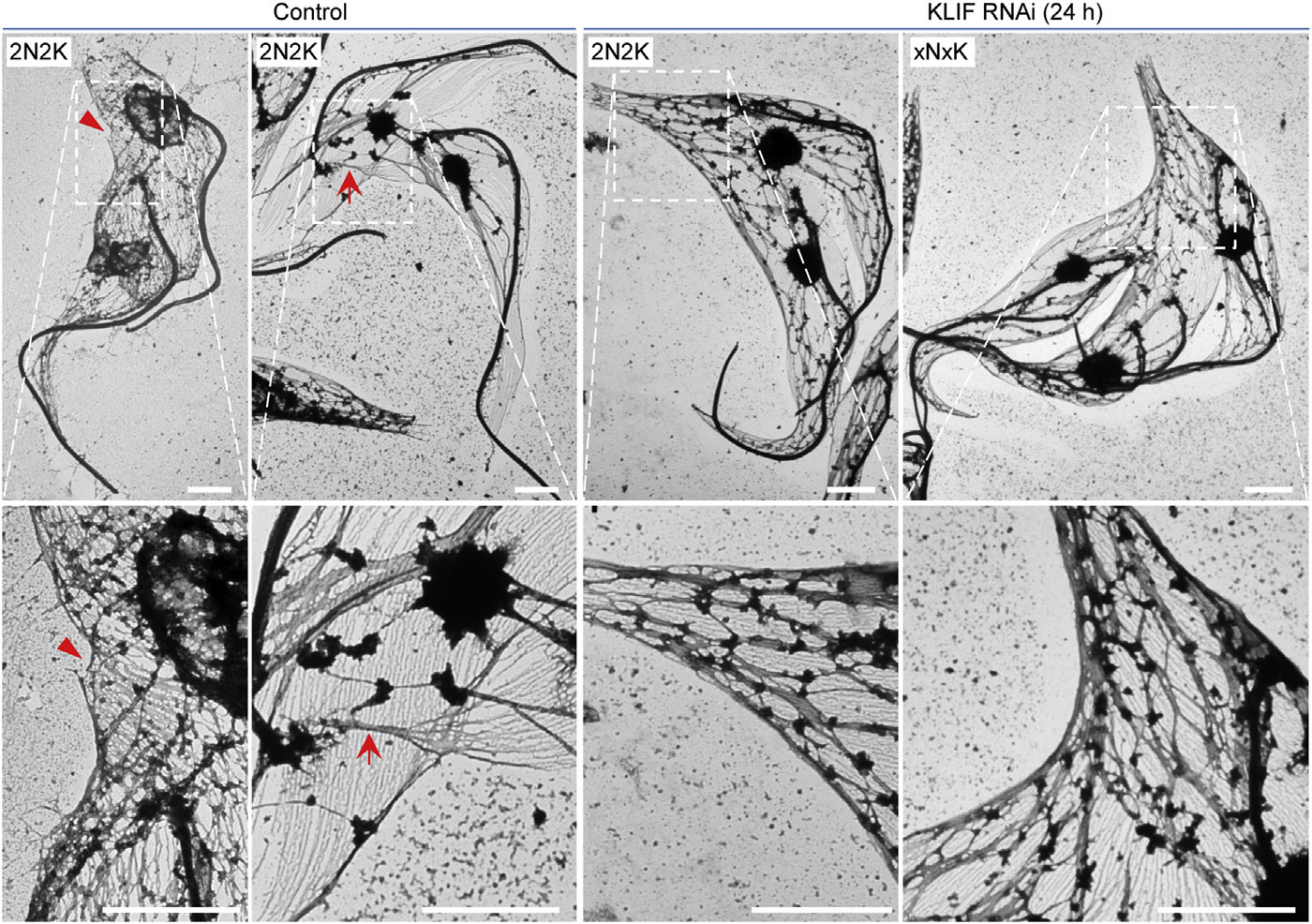
**KLIF RNAi inhibited microtubule bundling at the nascent posterior of the old-flagellum daughter cell**. Shown are TEM images of a binucleated (2N2K) cell from the noninduced control and KLIF RNAi-induced cell population and a multinucleated (3N3K) cell from KLIF RNAi-induced cell population. The *red arrowheads* indicate bundled microtubules at the nascent posterior of the OFD of a binucleated cell, and the *red arrows* indicate the thin thread of bundled microtubules at the posterior end of the OFD, which connects to the NFD, of a dividing binuclated cell. Scale bars: 2 μm. KLIF, kinesin localized to the ingressing furrow.

Finally, we explored the defects of cleavage furrow ingression in the KLIF RNAi cells by transmission electron microscopic (TEM) analysis of the detergent-extracted cytoskeleton to examine the microtubules along the ingressing cleavage furrow. We found that the microtubules along the ingressing cleavage furrow in the KLIF RNAi cells were not distorted (Fig. 2), suggesting that it was not the defect in the microtubules along the cleavage furrow that stalled cleavage furrow ingression in the KLIF-deficient cells. Thus, we examined the microtubules at the nascent posterior of the OFD cell of the dividing cells, as the nascent posterior represents the end of the cleavage furrow in trypanosomes. In the noninduced control 2N2K cells prior to cytokinesis initiation, the microtubules at the nascent posterior of the OFD cell started to form bundles, resulting in the protrusion of a subset of microtubules that would form the posterior of the OFD cell (Fig. 2, *red arrowhead*). At late stages of cytokinesis, the microtubules of the nascent posterior of the OFD cell were further bundled together, forming a thread of tightly bundled microtubules that connected the OFD cell to the NFD cell (Fig. 2, *red arrow*). Such microtubule bundling at the nascent posterior of the OFD cell of a dividing binucleated (2N2K) cell at late stages of cytokinesis was observed in 36 cells (∼97%) of the 37 cells viewed under TEM. However, the microtubules located at the nascent posterior of the OFD cell of the binucleated (2N2K) cells and multinucleated (xNxK) cells apparently failed to form bundles in 94 cells (∼96%) out of the 98 cells examined under TEM and, hence, there was no formation of the posterior end for the OFD cell (Fig. 2). These findings suggest that knockdown of KLIF disrupted microtubule bundling at the nascent posterior end of a dividing cell, thereby preventing the further ingression of the cleavage furrow toward the nascent posterior, leading to stalled furrow ingression.

### KLIF-, CIF1-, and FPRC-proximal proteins share a common set of CAPs

**Figure 3:**
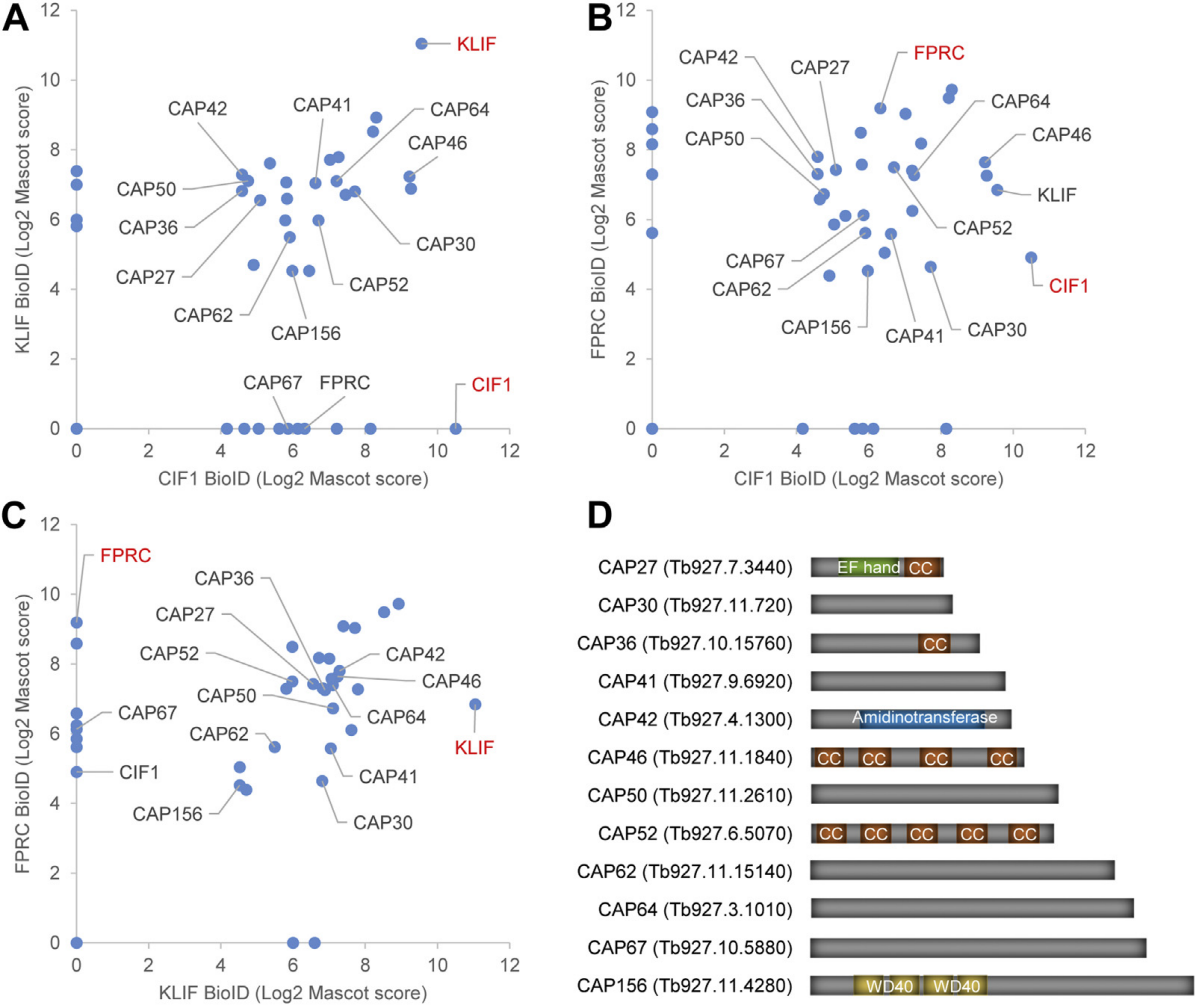
**Identification of KLIF-, CIF1-, and FPRC-proximal proteins by BioID**. *A*, KLIF- and CIF1-proximal proteins identified by BioID. *B*, CIF1- and FPRC-proximal proteins identified by BioID. *C*, KLIF- and FPRC-proximal proteins identified by BioID. *D*, schematic drawing of the cytoskeleton-associated proteins (CAPs) identified by KLIF, CIF1, and FPRC BioID. CC, coiled coil; CIF1, cytokinesis initiation factor 1; FPRC, FAZ tip-localized protein required for cytokinesis; KLIF, kinesin localized to the ingressing furrow; WD40, WD40-repeats domain.

To identify the potential KLIF-associated proteins that may function in controlling the ingression of the cleavage furrow, we carried out proximity-dependent BioID by expressing the BirA∗-fused KLIF from an ectopic locus in *T. brucei*, purifying biotinylated proteins through affinity chromatography and then analyzing the purified biotinylated proteins by mass spectrometry (LC-MS/MS). The proteins thus identified were searched against the *T. brucei* proteome, and those known contaminates, such as ribosomal proteins, metabolic enzymes, *etc*, were removed from the list of candidate KLIF-proximal proteins. Since KLIF localizes to the anterior tip of the NFD cell (or the new FAZ tip) during telophase and to the cleavage furrow during cytokinesis (12, 16), we reasoned that the KLIF-proximal proteins should also localize to these subcellular locations. Therefore, the remaining candidate KLIF-proximal proteins were searched for their subcellular localizations, either from the published work or from the TriTrypDB subcellular localization dataset released from the TrypTag project (25). All of the proteins that do not localize to the FAZ, the FAZ tip, the hook complex (as it overlaps with the new FAZ tip during early S phase), the cleavage furrow, and the cytoskeleton were excluded from the list of candidate KLIF-proximal proteins. The inclusion of cytoskeletal proteins as putative KLIF-associated proteins is due to the fact that the FAZ tip and the cleavage furrow may make contact with the cytoskeleton and that the microtubule cytoskeleton is known to play important roles in cytokinesis. We previously performed the BioID experiments with CIF1 and FPRC as bait (12, 14, 17), and we compared the final list of KLIF-proximal proteins with that of CIF1-proximal proteins and FPRC-proximal proteins so that we may identify a common set of proteins that are likely to function in the same cytokinesis regulatory pathway. Although a number of known cytokinesis regulators, such as CIF2, CIF3, CIF4, KPP1, FRW1, KAT60a, and KAT80, were identified by CIF1 and FPRC BioID, they were not identified by KLIF BioID (Table S1). However, a cohort of cytoskeleton-localized proteins were identified by all three BioID experiments (Fig. 3, *A*–*C* and Table S1), including TbAIR9, a CAP involved in determining the position of the cleavage furrow (21), and the two PAVE proteins that form the PAVE1–PAVE2 complex and play roles in posterior cytoskeleton remodeling (26). Since cytoskeleton remodeling occurs along the ingressing cleavage furrow during cytokinesis in *T. brucei*, we reasoned that some of the cytoskeleton-localized proteins might play roles in controlling cleavage furrow ingression and/or positioning. Therefore, functional characterization of these proteins might identify crucial cytokinesis regulators and provide insights into the control mechanism of cleavage furrow positioning and ingression in *T. brucei*.

Among the CAPs identified by BioID experiments with KLIF, CIF1, and FPRC as bait (Fig. 3, *A*–*C*), three proteins were recently named CAP42, CAP50, and CAP52 and were found to be required for maintaining cell morphology and for cytokinesis (22). We thus named the remaining CAPs as CAP27-CPA156 for CAP of XXX kDa (Fig. 3*D*). Six of these CAPs (CAP30, CAP41, CAP50, CAP62, CAP64, and CAP67) do not contain any detectable structural motifs, three of the CAPs (CAP36, CAP46, and CAP52) contain one or more coiled-coil (CC) motifs, and the remaining three CAPs contain either a putative amidinotransferase domain (CAP42), four WD40 repeats domain (CAP156), or an EF hand motif and a CC motif (CAP27) (Fig. 3*D*). The presence of different or no structural motifs suggests diverse functions for these CAPs. To examine whether these CAPs localize to any specific subdomains of the cytoskeleton, we tagged each of these CAPs with a triple HA epitope from one of their endogenous loci and performed immunofluorescence microscopy. Six CAP proteins (CAP27, CAP36, CAP41, CAP42, CAP50, and CAP64) were distributed to the entire cytoskeleton, two CAP proteins (CAP46 and CAP52) appeared to localize to the anterior portion and the dorsal edge of the cell, two CAP proteins (CAP30 and CAP67) appeared to localize to the anterior portion of the cell, one CAP protein (CAP62) localized to the hook complex region, and one CAP protein (CAP156) localized to the entire cytoskeleton but was enriched at the posterior end during G1 to mitosis and at the nascent posterior end during telophase (Fig. S2*A*). The localization of these CAP proteins to different subdomains of the cytoskeleton indicates that they may play distinct functions. To confirm that these CAPs associate with cytoskeleton, we prepared the cytosolic and cytoskeletal fractions of the cells expressing each of the 12 CAP proteins tagged with a triple HA epitope and then performed Western blotting to monitor their distribution in the two fractions. With the only exception of CAP27, which was detected in the cytosolic fraction for a small amount of protein and in the cytoskeletal fraction for most of protein, all other CAPs were only detected in the cytoskeletal fraction (Fig. S2*B*). Notably, three separate bands ranging from ∼45 to 65 kDa were detected for CAP62 (Fig. S2*B*), of which the two lower bands may represent truncated products. Nonetheless, these results confirmed that the 12 CAP proteins associated with the cytoskeleton of trypanosome cells.

### CAP50, CAP42, CAP27, CAP36, and CAP156 are required for cleavage furrow ingression

We performed RNAi to ablate the expression of each of the 12 CAP proteins identified by KLIF, CIF1, and FPRC BioID experiments in the procyclic form of *T. brucei*, and seven out of the 12 CAP proteins were found to be involved in cytokinesis. In this and the following sections, we performed phenotypic analyses of the RNAi cells and investigated the specific defects on cytokinesis caused by the depletion of each of these CAP proteins by light microscopy and electron microscopy.

**Figure 4:**
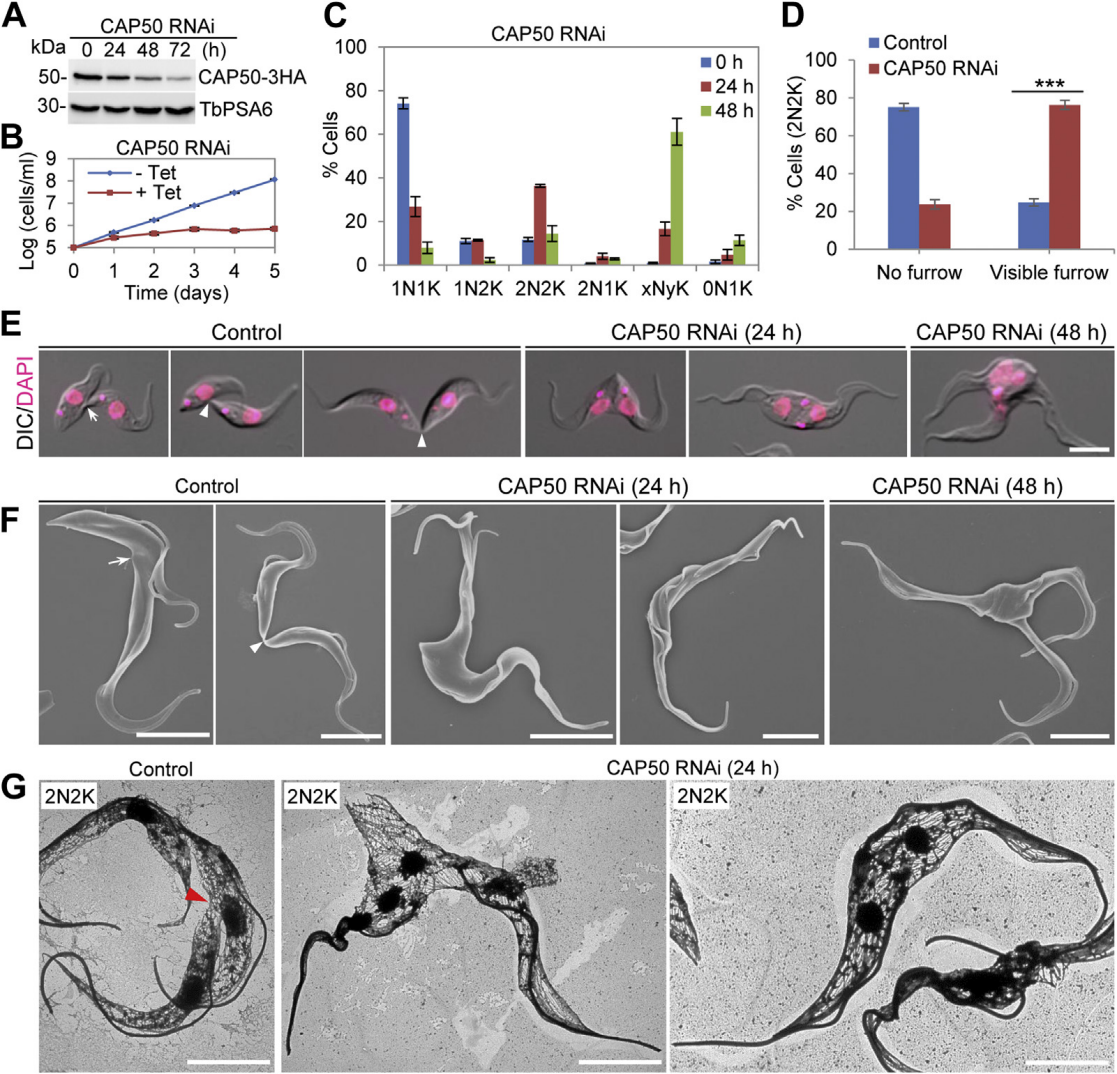
**CAP50 is required for cleavage furrow ingression**. *A*, Western blotting to detect CAP50 protein level before and after RNAi induction. CAP50 was endogenously tagged with a triple HA epitope and detected by anti-HA antibody. TbPSA6 served as a loading control. *B*, CAP50 RNAi inhibited cell proliferation. *C*, effect of CAP50 RNAi on cell cycle progression. Shown is the quantitation of cells at different cell cycle stages before and after CAP50 RNAi induction. Two hundred cells for each time point were counted for the number of nucleus (N) and kinetoplast (K), and error bars indicate SD from three independent experiments. *D*, CAP50 RNAi inhibited cleavage furrow ingression. Shown is the quantitation of binucleated (2N2K) cells without or with a visible cleavage furrow before and after CAP50 RNAi for 24 h. Two hundred cells were counted for each time point, and error bars indicate SD from three independent experiments. ∗∗∗*p* < 0.001. *E* and *F*, the control and CAP50 RNAi cells undergoing cytokinesis imaged by light microscopy (*E*) and scanning electron microscopy (*F*). The *white arrow* indicates the nascent posterior of the old-flagellum daughter cell of a dividing cell, whereas the *white arrowheads* indicate the cytoplasmic bridge connecting the old-flagellum daughter cell to the ventral side or the posterior end of the new-flagellum daughter cell. Scale bars: 5 μm. *G*, transmission electron microscopic analysis of negatively stained cytoskeleton of control and CAP50 RNAi-induced cells. The *red arrowhead* indicates the nascent posterior end. Scale bars: 5 μm.

Among the seven RNAi cell lines, CAP50 RNAi cell line showed the strongest growth defects (see below); therefore, we first presented the functional characterization of CAP50. Western blotting showed the gradual decrease of the protein level of CAP50, which was endogenously tagged with a triple HA epitope (Fig. 4*A*). This downregulation of CAP50 protein resulted in severe growth defects by inhibiting cell proliferation after 2 days of RNAi induction (Fig. 4*B*). Further analysis of cell cycle progression by counting the cells with different numbers of nucleus and kinetoplast showed that there was an increase of binucleated (2N2K) cells from ∼12% to ∼36% after 24 h of RNAi and then multinucleated (xNyK, x > 2, y ≥ 1) cells to ∼61% of the total population after 48 h of RNAi induction (Fig. 4*C*), indicating defective cytokinesis. There was also the emergence of cells with either two nuclei and one kinetoplast (2N1K) or only a kinetoplast (0N1K) (Fig. 4*C*), indicating that aberrant cytokinesis might have occurred for 2N2K cells to divide into 2N1K and 0N1K cells. We next asked which cytokinesis stage was impaired after CAP50 knockdown by examining the cleavage furrow of the binucleated cells, and the results showed that after 24 h of CAP50 RNAi, the binucleated cells with a visible cleavage furrow were increased from ∼25% to ∼76% (Fig. 4*D*), demonstrating that CAP50 knockdown inhibited cleavage furrow ingression.

We next performed light microscopy and SEM to examine the dividing cells from the noninduced control and the CAP50 RNAi-induced population. In the noninduced control cells, during early cytokinesis stages, the nascent posterior of the OFD cell of a dividing binucleated cell is formed at the ventral side of the NFD cell (Fig. 4, *E* and *F*, arrows), and during late cytokinesis stages, the posterior tip of the OFD cell is connected to the ventral side of the NFD cell or the posterior tip of the NFD cell (Fig. 4, *E* and *F*, arrowheads). In the CAP50 RNAi-induced cells, however, the nascent posterior of the OFD cell of a dividing binucleated cell was not evidently formed or was not resolved from the posterior of the NFD cell (Fig. 4, *E* and *F*). In the CAP50 RNAi-induced multinucleated cells, multiple well-separated anterior ends were found, with the posteriors of the multiple daughter cells unseparated or fused together (Fig. 4, *E* and *F*). We next performed TEM analysis of detergent-extracted cytoskeleton to examine the cytoskeletal microtubules at the nascent posterior of the OFD cell. In control cells, the microtubules at the nascent posterior of the OFD cell of a dividing binucleated cell started to form bundles (Fig. 4*G*, red arrowhead). However, in the CAP50 RNAi-induced cells, due to the lack of formation of the nascent posterior of the OFD cell in a dividing binucleated cell, microtubule bundling was not observed (Fig. 4*G*). These results suggest that CAP50 is involved in the formation of the nascent posterior of the OFD cell during cytokinesis to ensure cleavage furrow ingression.

**Figure 5:**
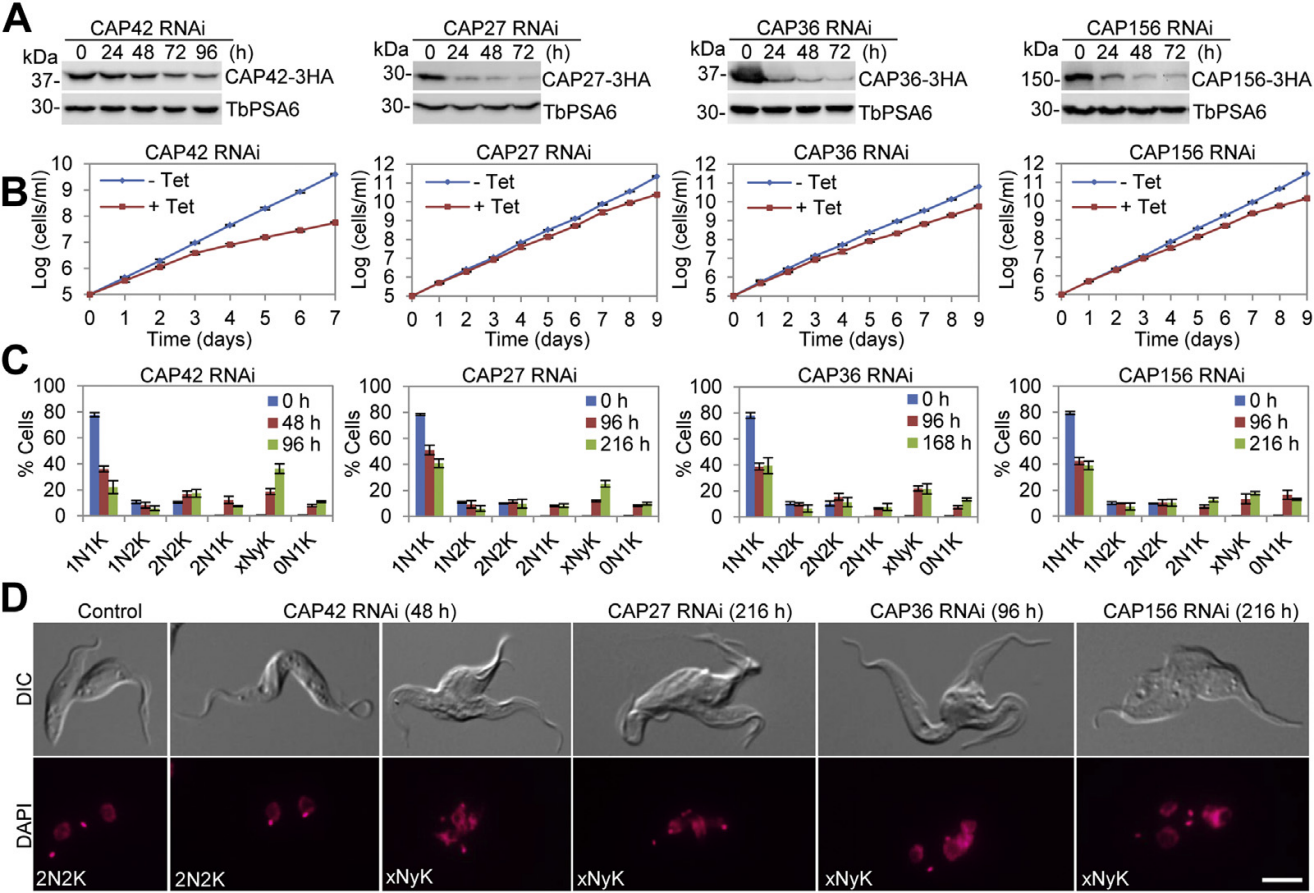
**CAP42, CAP27, CAP36, and CAP156 are each required for cleavage furrow ingression.***A*, Western blotting to detect the protein levels of CAP42, CAP27, CAP36, and CAP156 before and after RNAi induction. CAP42, CAP27, CAP36, and CAP156 were each endogenously tagged with a triple HA epitope in their respective RNAi cell line. *B*, effect of CAP42, CAP27, CAP36, and CAP156 RNAi on cell proliferation. *C*, effect of CAP42 RNAi, CAP27 RNAi, CAP36 RNAi, and CAP156 RNAi on cell cycle progression. Shown is the quantitation of cells of different nuclear and kinetoplast configurations before and after RNAi induction. Two hundred cells for each time point were counted, and error bars indicate SD. from three independent experiments. *D*, a noninduced control cell and a CAP42 RNAi cell of the 2N2K configuration undergoing cytokinesis, and a CAP42 RNAi-induced cell, a CAP27 RNAi-induced cell, a CAP36 RNAi-induced cell, and a CAP156 RNAi-induced cell of the xNyK configuration undergoing cytokinesis imaged by light microscopy. Scale bar: 5 μm.

We next characterized the phenotype caused by knockdown of CAP27, CAP36, CAP42, and CAP156, as depletion of each of the four proteins also caused defects in cell proliferation. We first confirmed the knockdown of the four CAP proteins by Western blotting with anti-HA antibody to detect the expression level of these CAP proteins, which were each tagged with a triple HA epitope from their respective endogenous locus (Fig. 5*A*). Knockdown of CAP42 by RNAi resulted in more severe growth defects than other three knockdowns (Fig. 5*B*). Quantitative analysis of the cell cycle progression before and after CAP42 RNAi induction showed that after 96 h of RNAi induction, cells of the 1N1K and 1N2K configurations were gradually decreased from ∼78% to ∼22% and from ∼11% to ∼6%, respectively, which was followed by the gradual increase of cells with 2N2K and xNyK (x > 2, y ≥ 1) configurations from ∼10% to ∼17% and from <1% to ∼36%, respectively (Fig. 5*C*), indicating that cytokinesis was compromised. Notably, there was also the gradual accumulation of cells of abnormal configurations, such as 2N1K and 0N1K cells (Fig. 5*C*), which could be derived from aberrant cytokinesis of the 2N2K cells. We next performed light microscopic analysis of the binucleated (2N2K) and multinucleated (xNyK, x > 2, y ≥ 1) cells, and we found that, like those cells depleted of CAP50, the binucleated cells after CAP42 RNAi induction either did not form the nascent posterior or failed to resolve the nascent posterior, causing the cells being stuck at the late stage of cytokinesis (Fig. 5*D*). Similarly, those multinucleated cells collected from CAP42 RNAi-induced population were found to contain multiple cleavage furrows, and the posteriors of the two daughter cells were fused together or were not separated (Fig. 5*D*). These results indicated that RNAi of CAP42 led to stalled furrow ingression and inhibited cytokinesis completion.

Unlike knockdown of CAP50 and knockdown of CAP42, however, knockdown of CAP27, CAP36, and CAP156 each caused moderate growth defects (Fig. 5*B*). Quantitation of cells with different numbers of nucleus and kinetoplast from the three RNAi cell lines showed that there was the accumulation of xNyK (x > 2, y ≥ 1) cells to ∼25%, ∼22%, and ∼18% for CAP27 RNAi, CAP36 RNAi, and CAP156 RNAi, respectively (Fig. 5*C*), indicating that cytokinesis was defective in these RNAi cell lines. Further examination of the xNyK cells from each of the three RNAi cell lines showed that these cells possessed multiple cleavage furrows (Fig. 5*D*), similar to that observed in CAP50 RNAi cells (Fig. 4, *E* and *F*) and CAP42 RNAi cells (Fig. 5*D*). Together, these results suggest that CAP27, CAP36, and CAP156 are each required for cleavage furrow ingression in the procyclic form of *T. brucei*.

### CAP46 and CAP52 form a complex and are required for positioning the cleavage furrow

**Figure 6:**
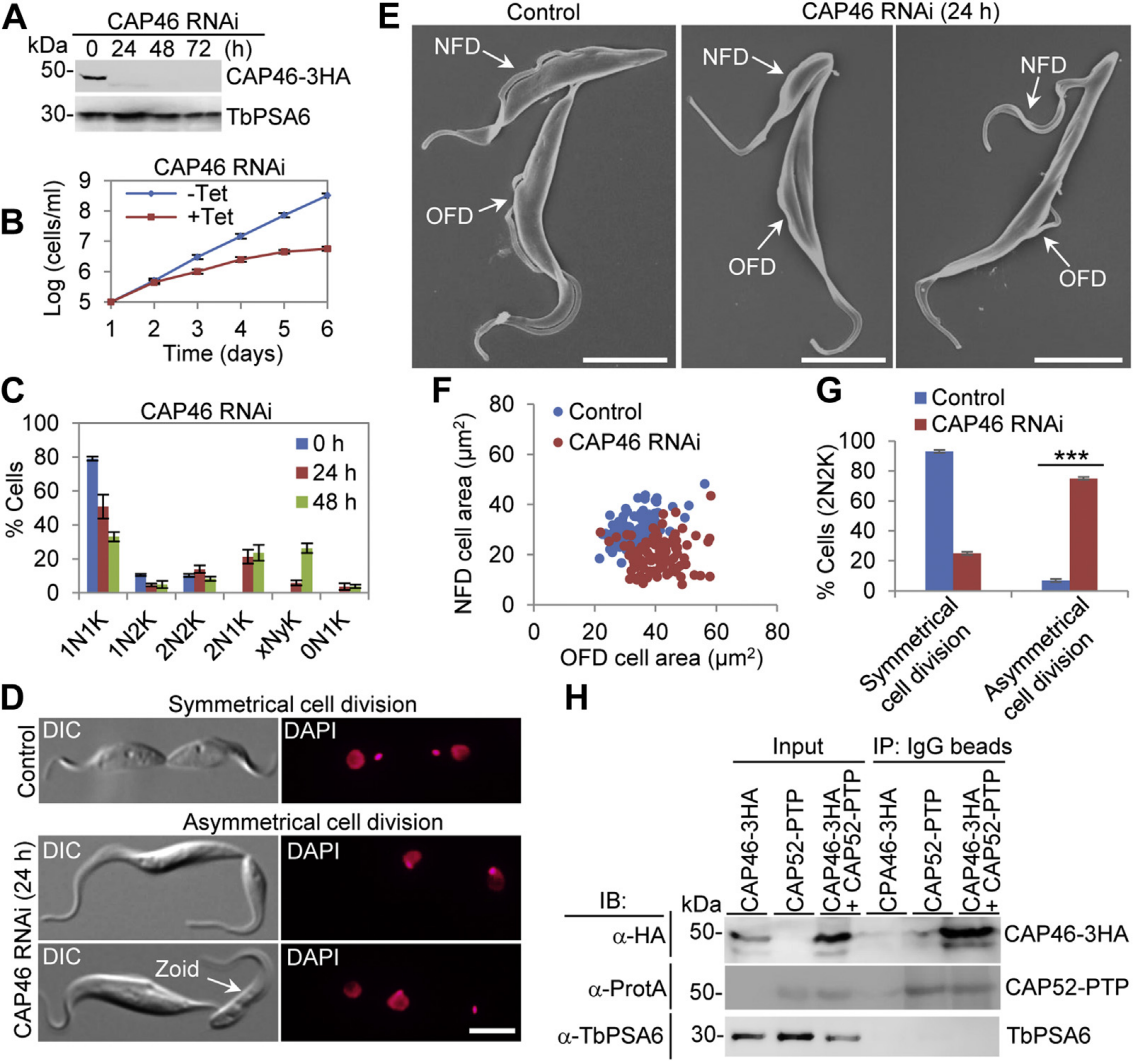
**CAP46 is required for cleavage furrow positioning**. *A*, Western blotting to detect CAP46 protein level before and after CAP46 RNAi induction. Endogenous 3HA-tagged CAP46 was detected by anti-HA antibody. TbPSA6 served as a loading control. *B*, CAP46 RNAi caused severe growth defects. *C*, effect of CAP46 RNAi on cell cycle progression. Shown is the quantitation of cells at different cell cycle stages before and after CAP46 RNAi. Two hundred cells for each time point were counted, and error bars indicate SD from three independent experiments. *D* and *E*, visualization of control cells undergoing symmetrical cytokinesis and CAP46 RNAi-induced cells undergoing asymmetrical cytokinesis by light microscopy (*D*) and scanning electron microscopy (*E*). NFD, new-flagellum daughter; OFD, old-flagellum daughter. Scale bars: 5 μm. *F*, measurement of the size of the new-flagellum daughter (NFD) and old-flagellum daughter (OFD) of dividing binucleated (2N2K) cells before and after CAP46 RNAi induction for 24 h. *G*, quantitation of cells undergoing symmetrical or asymmetrical cytokinesis before and after CAP46 RNAi induction for 24 h. Two hundred cells for each time point were counted, and error bars indicate SD from three independent experiments. ∗∗∗*p* < 0.001. *H*, *in vivo* interaction between CAP46 and CAP52 detected by co-immunoprecipitation. IB, immunoblotting; IP, immunoprecipitation.

The localization of CAP46 to the dorsal edge and the anterior portion of the cell raised a question about whether it is involved in cytokinesis. To test this possibility, we investigated the function of CAP46 by RNAi in the procyclic form. Western blotting confirmed the depletion of CAP46, which was endogenously tagged with a triple HA epitope, after RNAi induction for 24 h (Fig. 6*A*). This depletion of CAP46 caused severe growth defects (Fig. 6*B*), demonstrating the requirement of CAP46 for cell proliferation. Analysis of the cell cycle by counting the cells with different numbers of nucleus (N) and kinetoplast (K) showed that abnormal 2N1K cells and xNyK (x > 2, y ≥ 1) cells emerged after CAP46 RNAi induction (Fig. 6*C*), indicating defects in kinetoplast segregation and cytokinesis. When examining the binucleated cells, we found that CAP46 RNAi caused asymmetrical division of binucleated cells, which resulted in the production of a smaller NFD cell containing either one nucleus and one kinetoplast or only one kinetoplast (termed zoid cell) (Fig. 6*D*). SEM analysis of CAP46 RNAi cells confirmed the asymmetrical cell division (Fig. 6*E*), and quantitation of the size of the NFD and OFD cells confirmed that the size of the NFD cell was significantly smaller than that of the OFD cell (Fig. 6*F*). The average size of the NFD cells was reduced from ∼30 μm^2^ to ∼20 μm^2^, whereas the average size of the OFD cells was increased from ∼33 μm^2^ to ∼40 μm^2^ after CAP46 RNAi induction for 24 h (Fig. 6*F*). The binucleated cells undergoing asymmetrical division accounted for ∼75% of the binucleated cell population after CAP46 RNAi induction for 24 h (Fig. 6*G*). Thus, CAP46 appears to play a role in determining the position of the cleavage furrow.

Since both CAP46 and CAP52 localize to the dorsal edge and the anterior portion of the cell, we tested whether the two proteins formed a complex *in vivo* in *T. brucei*. We generated a cell line that co-expresses CAP46 tagged endogenously with a triple HA epitope and CAP52 tagged endogenously with a PTP epitope. As controls, we also generated a cell line that expresses CAP46-3HA and a cell line that expresses CAP52-PTP. Immunoprecipitation (IP) of CAP52-PTP from *T. brucei* cell lysate, which was prepared by sonication to solubilize both proteins, was able to pull down CAP46-3HA (Fig. 6*H*), demonstrating that CAP46 and CAP52 form a complex that might cooperate to regulate cleavage furrow positioning.

**Figure 7:**
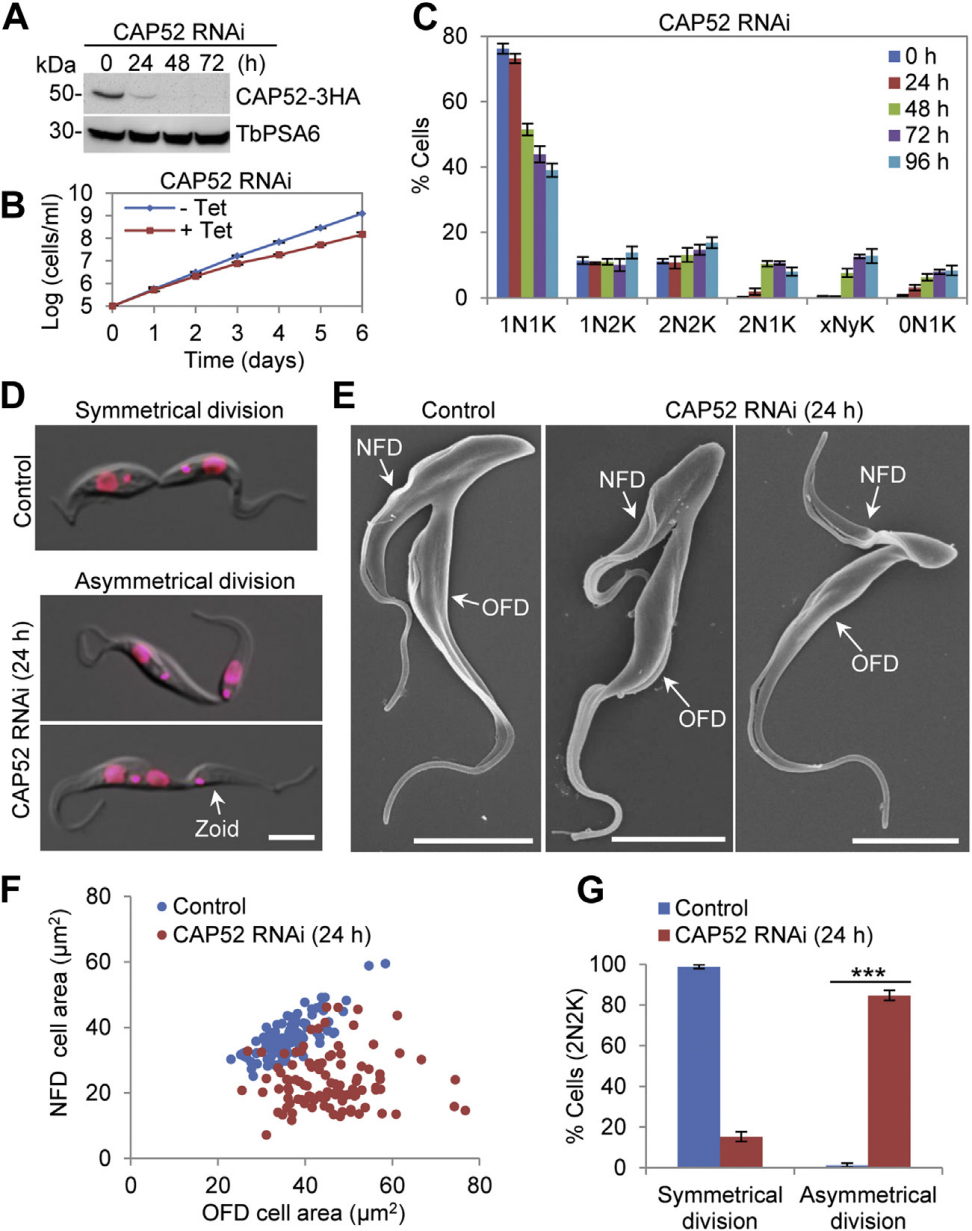
**CAP52 is required for cleavage furrow positioning**. *A*, Western blotting to detect CAP52 protein level before and after CAP52 RNAi induction. Endogenous 3HA-tagged CAP52 was detected by anti-HA antibody. TbPSA6 served as a loading control. *B*, RNAi of CAP52 caused moderate growth defects. *C*, effect of CAP52 RNAi on cell cycle progression. Shown is the quantitation of cells at different cell cycle stages before and after CAP46 RNAi. Two hundred cells for each time point were counted, and error bars indicate SD from three independent experiments. *D* and *E*, visualization of control cells undergoing symmetrical cytokinesis and CAP52 RNAi-induced cells undergoing asymmetrical cytokinesis by light microscopy (*D*) and scanning electron microscopy (*E*). NFD, new-flagellum daughter; OFD, old-flagellum daughter. Scale bars: 5 μm. *F*, measurement of the size of the new-flagellum daughter (NFD) and old-flagellum daughter (OFD) of dividing binucleated (2N2K) cells before and after CAP52 RNAi induction for 24 h. *G*, quantitation of cells undergoing symmetrical or asymmetrical cytokinesis before and after CAP52 RNAi induction for 24 h. Two hundred cells for each time point were counted, and error bars indicate SD from three independent experiments. ∗∗∗*p* < 0.001.

To test whether CAP52 plays a similar role as CAP46 in positioning the cleavage furrow, we investigated the function of CAP52 by RNAi in the procyclic form of *T. brucei*. Knockdown of CAP52 was confirmed by Western blotting, which showed gradual depletion of CAP52-3HA after RNAi induction for 24 h (Fig. 7*A*). Depletion of CAP52 caused moderate growth defects (Fig. 7*B*), and like CAP46 RNAi, CAP52 RNAi also led to the emergence of abnormal 2N1K and xNyK cells (Fig. 7*C*), suggesting defective kinetoplast segregation and cytokinesis. Additionally, there was also the emergence of anucleate (0N1K) cells to ∼7 to 8% of the total population after RNAi induction for 72 to 96 h (Fig. 7*C*), indicating that aberrant cytokinesis occurred for 2N2K cells to produce 2N1K cells and 0N1K cells. We next examined the 2N2K cells, which were slightly increased after CAP52 RNAi induction (Fig. 7*C*), by light microscopy and scanning electron microscopy, and found that CAP52 RNAi also caused asymmetrical cell division of 2N2K cells by producing smaller sized NFD cells containing either one nucleus and one kinetoplast or one kinetoplast (Fig. 7, *D*–*F*). The average size of the NFD cells was reduced from ∼30 μm^2^ to ∼22 μm^2^, whereas the average size of the OFD cells was increased from ∼31 μm^2^ to ∼38 μm^2^ (Fig. 7*F*). The 2N2K cells that were undergoing asymmetrical cell division accounted for ∼85% of the total 2N2K population after CAP52 RNAi induction for 24 h (Fig. 7*G*). Together, these results suggest that CAP52 is involved in the control of the positioning of the cleavage furrow by cooperating with CAP46 at the anterior portion and the dorsal edge of the cytoskeleton of a trypanosome cell.

### CAP46 and CAP52 are interdependent for maintaining their protein stability

**Figure 8:**
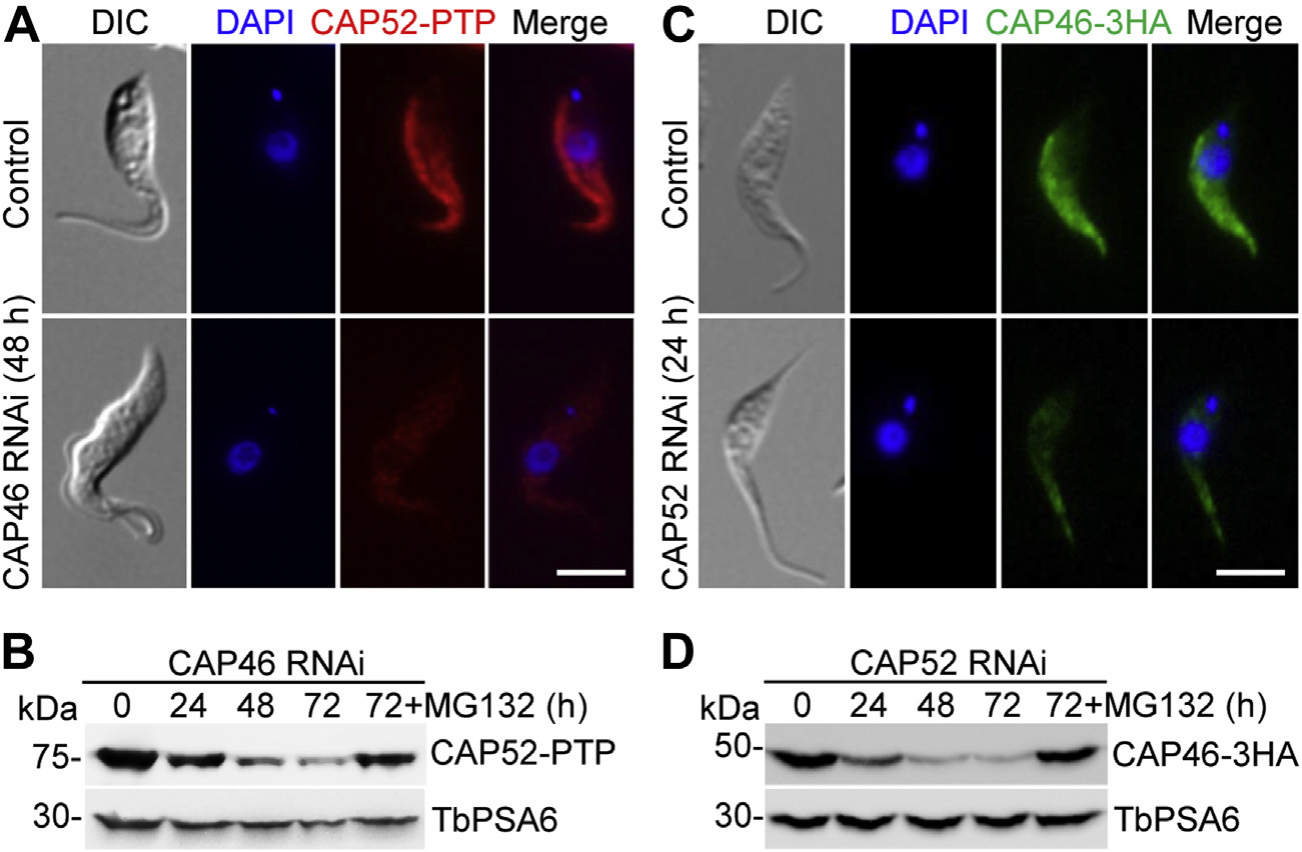
**CAP46 and CAP52 are interdependent for maintaining their stability**. *A*, effect of CAP46 RNAi on CAP52 localization. CAP46 RNAi cell line expressing endogenously PTP-tagged CAP52 was induced for RNAi, and cells were immunostained with anti-Protein A antibody. Scale bar: 5 μm. *B*, Western blotting to detect CAP52 protein level in noninduced and RNAi-induced cells of the CAP46 RNAi cell line. CAP52 was endogenously tagged with PTP and detected by anti-Protein A antibody. TbPSA6 served as a loading control. MG-132 was added to the RNAi-induced cells at 64 h after induction and incubated for 8 h. *C*, effect of CAP52 RNAi on CAP46 localization. CAP46 was endogenously tagged with a triple HA epitope in the CAP52 RNAi cell line and detected by FITC-conjugated anti-HA antibody. Scale bar: 5 μm. *D*, effect of CAP52 RNAi on CAP46 protein stability. MG-132 was added after RNAi induction for 64 h and incubated for 8 h. TbPSA6 served as a loading control.

Since CAP46 and CAP52 interact *in vivo* (Fig. 6*H*), we investigated the potential functional interplay between them by examining the localization of one protein in cells depleted of the other protein. Immunofluorescence microscopy of noninduced and CAP46 RNAi-induced cells expressing endogenously PTP-tagged CAP52 showed that after CAP46 RNAi induction, CAP52 protein was no longer enriched at the anterior portion and the dorsal edge of the cell and the overall protein level of CAP52 appeared to be significantly lower than that in the noninduced control cell (Fig. 8*A*). This notion was confirmed by Western blotting, which showed that the level of CAP52 protein was gradually decreased after CAP46 RNAi induction, and when the proteasome inhibitor MG-132 was added to the CAP46 RNAi cells, CAP52 protein level was elevated (Fig. 8*B*), indicating that CAP46 RNAi caused the destabilization of CAP52. Conversely, CAP52 RNAi also disrupted the enrichment of CAP46 and destabilized CAP46 (Fig. 8, *C* and *D*). Together, these results suggest that CAP46 and CAP52 are interdependent for maintaining their protein stability.

### Effect of CAP50 and CAP46 knockdown on KLIF localization

**Figure 9:**
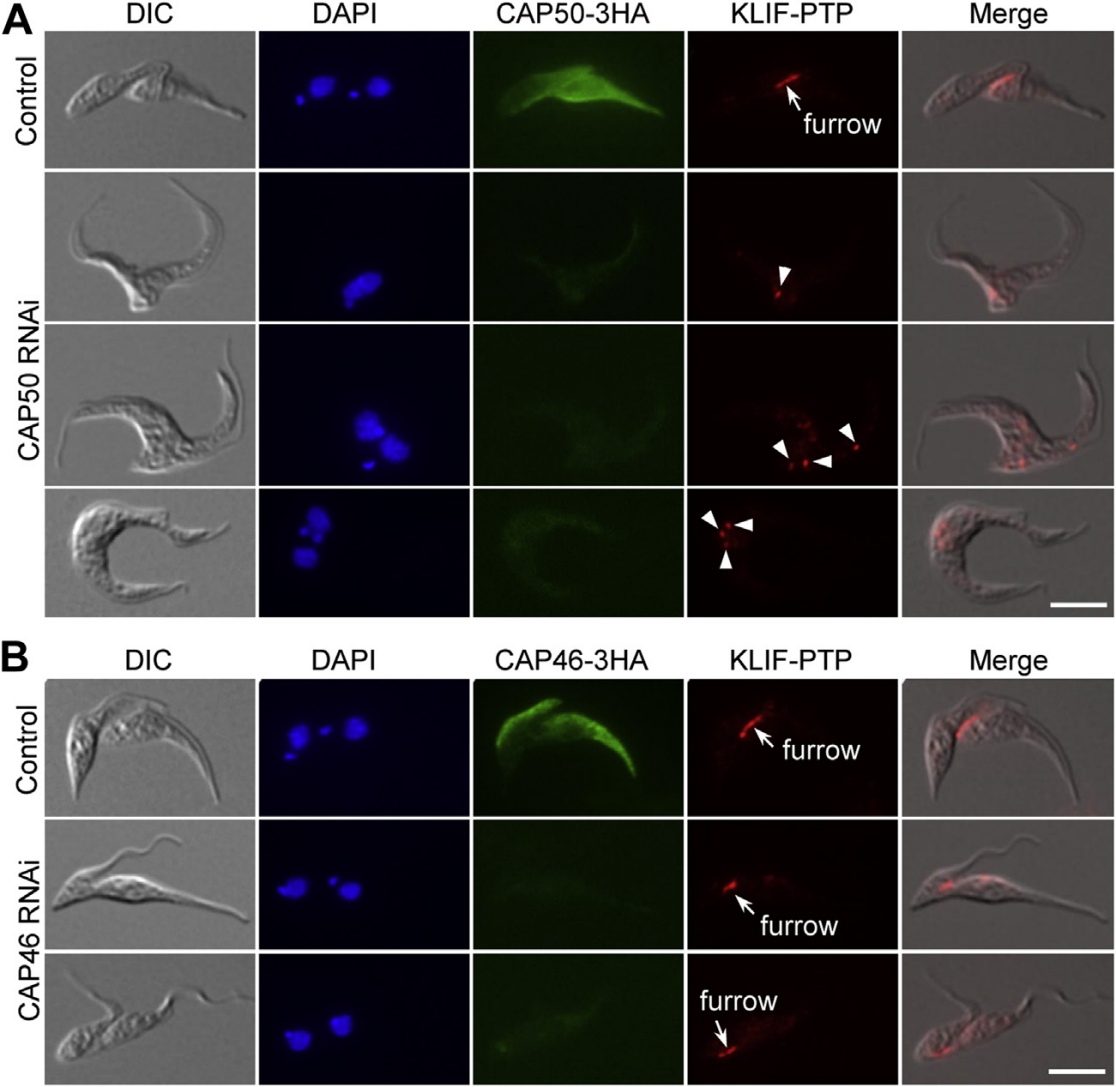
**Effect of CAP50 RNAi and CAP46 RNAi on KLIF localization.***A*, subcellular localization of KLIF, endogenously tagged with a PTP epitope, in noninduced control and CAP50 RNAi-induced cells and in noninduced control and CAP46 RNAi-induced cells (*B*). The *arrow* indicates KLIF signal at the ingressing cleavage furrow, and the *arrowheads* indicate KLIF signal outside of the cleavage furrow. Scale bars: 5 μm. KLIF, kinesin localized to the ingressing furrow.

The identification of CAP proteins as KLIF-proximal proteins and their essential roles in cytokinesis prompted us to investigate the potential effect of their deficiency on KLIF localization during cytokinesis. We chose the CAP50 RNAi cell line and the CAP46 RNAi cell line as representatives for this investigation because they represented two distinct cytokinesis defects reported in this work: the defective furrow ingression (Fig. 4) and the defective furrow positioning (Fig. 6). To this end, we tagged KLIF with a C-terminal PTP epitope from one of its endogenous loci in the CAP50 RNAi cell line expressing endogenously 3HA-tagged CAP50 and the CAP46 RNAi cell line expressing endogenously 3HA-tagged CAP46 (Fig. 9). Immunofluorescence microscopy was then performed, and binucleated cells that were undergoing cytokinesis were examined for the localization of KLIF before and after RNAi induction. In the noninduced control cells, KLIF was detected at the ingressing cleavage furrow (Fig. 9, *A* and *B*). In the binucleated cells depleted of CAP50, KLIF was detected as either one or multiple fluorescence foci within the posterior portion of the cell body in all (>200) of the cells examined (Fig. 9*A*). However, in the binucleated cells depleted of CAP46, KLIF was still detectable at the ingressing cleavage furrow that was positioned between the two asymmetrically dividing daughter cells (Fig. 9*B*). These results suggest that depletion of CAP50, but not CAP46, disrupted KLIF localization to the cleavage furrow.

## Discussion

*T. brucei* is known to employ an actomyosin-independent mechanism for cleavage furrow ingression, although the underlying mechanism remains poorly understood. Several proteins have been found to localize to the ingressing cleavage furrow, including Aurora B kinase and its associated chromosomal passengers TbCPC1 and TbCPC2 (9, 10), the trypanosome-specific cytokinesis regulators CIF1, CIF3, CIF4, FRW1, and KLIF (11, 12, 13, 15, 16, 17), of which only FRW1 and KLIF emerge at the new FAZ tip (or the anterior tip of the NFD cell or the cytokinesis initiation site) immediately prior to the ingression of the cleavage furrow from the anterior tip of the NFD cell of a dividing binucleated cell (12, 16). Surprisingly, knockdown of FRW1 in procyclic trypanosomes had little effect on cytokinesis (19). It suggests that either FRW1 is nonessential for cytokinesis in the procyclic form or RNAi was unable to completely deplete FRW1 protein such that the remaining FRW1 protein is sufficient to promote cytokinesis. The previous work on KLIF showed that it was required for cytokinesis completion (16), but no effort was made to investigate the requirement of the KMD and the putative TPM domains for KLIF’s function in driving furrow ingression. Through genetic complementation, we demonstrated that the KMD, but not the putative TPM domains, was essential for KLIF to promote cleavage furrow ingression (Fig. 1). Tropomyosin is a CC motif-containing protein, and in the fission yeast, it is required for cytokinesis by forming part of the actomyosin contractile ring at the cleavage furrow (27). *T. brucei* does not express a homolog of tropomyosin, and given the lack of the actomyosin contractile ring machinery in *T. brucei*, the nonessentiality of the putative TPM domains of KLIF in cytokinesis appears to be consistent with the actomyosin-independent mechanism for *T. brucei* cytokinesis.

Through SEM and TEM analyses of the dividing cells collected from KLIF RNAi-induced cell population, we demonstrated that KLIF was required for microtubule bundling at the nascent posterior of the OFD cell to ensure cleavage furrow ingression to reach the nascent posterior tip of the OFD cell for a successful cell division (Figs. 1 and 2). Since KLIF is not required for the initiation of furrow ingression as well as for the early to the mid stages of furrow ingression, it suggests that KLIF is unlikely to function as a component of the molecular machinery that drives cleavage furrow ingression in *T. brucei*. Instead, KLIF likely functions to promote the remodeling of the microtubule cytoskeleton at the nascent posterior of the OFD cell to coordinate furrow ingression. A recent study on the biophysical properties of KLIF demonstrated that KLIF possessed the activity to bundle microtubules *in vitro* (Sladewski T.E. *et al*., Biorxiv, 2021, doi: https: doi.org/10.1101/2021.11.04.467292), which provided evidence to corroborate the observed defective microtubule bundling at the nascent posterior of the OFD cell caused by KLIF RNAi. Alternatively, since KLIF is a plus-ends directed motor protein (16), KLIF may function as a cargo transporter to transport certain proteins that are essential for microtubule bundling to the region of the nascent posterior of the OFD cell, thereby enabling these proteins to promote microtubule bundling for further ingression of the cleavage furrow and completion of cytokinesis.

For a better understanding of the roles of KLIF in cytokinesis, we aimed to identify the proteins that might interact with and cooperate with KLIF to promote furrow ingression by using the BioID approach. A cohort of CAPs was identified as the near neighboring proteins of KLIF, and all but one of them were also identified as near neighbor proteins of CIF1 and FPRC (Fig. 3), suggesting that these cytoskeletal proteins may play functions that are related to the three cytokinesis regulators. Indeed, several of these cytoskeletal proteins were previously reported to be required for cytokinesis (21, 22). In an attempt to comprehensively understand the potential roles of these cytoskeletal proteins in regulating cytokinesis, we characterized these KLIF-, CIF1-, and FPRC-associated cytoskeletal proteins and found that they localized to either the entire cytoskeleton or discrete subdomains of the cytoskeleton (Fig. S2) and that some of them played distinct functions in cytokinesis (Figure 4, Figure 5, Figure 6, Figure 7). Five CAP proteins, CAP50, CAP42, CAP27, CAP36, and CAP156, appear to be each required for the resolution of the nascent posterior during cytokinesis (Figs. 4 and 5), and they localize to the entire cytoskeleton, with the exception of CAP156, which is enriched at the nascent posterior region during cytokinesis (Fig. S2). It should be noted that knockdown of CAP27, CAP36, and CAP156 each caused only moderate cytokinesis defects (Fig. 5), which could be due to the partial knockdown of their respective proteins (Fig. 5*A*). Another possibility is that these three CAP proteins might play redundant functions; thus, the deficiency caused by knockdown of one of these CAP proteins might be compensated by other CAP protein(s). It is also possible that the cytokinesis defects caused by the knockdown of these three CAP proteins were due to an indirect effect by affecting their partner protein(s), which plays a direct role in cleavage furrow ingression. Nonetheless, the accumulation of multinucleated cells with unresolved posterior ends (Fig. 5*D*) suggests the involvement of these CAP proteins in late stages of cleavage furrow ingression. Finally, CAP46 and CAP52 are each required for maintaining the position of the cleavage furrow to ensure symmetrical cytokinesis (Figs. 6 and 7), and they localize to the anterior and the dorsal edge of the cell (Fig. S2). Although the mechanistic roles of these CAP proteins in regulating cytokinesis remain unclear, the requirement of certain CAPs for cytokinesis suggests the involvement of cytoskeleton remodeling, especially at the nascent posterior and along the cell division plane, in cleavage furrow placement and ingression.

The essential requirement of CAP50, but not CAP46, for the localization of KLIF to the cleavage furrow (Fig. 9) is consistent with the cytokinesis defects caused by the knockdown of these two proteins and with the function of KLIF in promoting cleavage furrow ingression during late stages of cytokinesis. Depletion of CAP50 caused defective cleavage furrow ingression and nascent posterior resolution at late stages of cytokinesis (Fig. 4), during which KLIF plays a crucial role in promoting cleavage furrow ingression (Figs. 1 and 2). Thus, the mislocalization of KLIF in CAP50-depelted cells (Fig. 9*A*) contributed, at least partly, to the defective furrow ingression and nascent posterior resolution. In contrast, depletion of CAP46 caused mispositioning of the cleavage furrow, but it did not inhibit the ingression of the cleavage furrow and the completion of cytokinesis, albeit in an asymmetrical manner (Fig. 6); both the ingression of the furrow and the completion of cytokinesis appear to depend on KLIF located at the cleavage furrow. Therefore, the localization of KLIF to the ingressing cleavage furrow in CAP46-depleted cells (Fig. 9*B*) apparently is required for the completion of the asymmetrical cytokinesis. Although the potential effect of the knockdown of other CAP proteins on KLIF localization remains to be determined, based on the cytokinesis defects caused by their knockdowns, we predict that CAP42, CAP27, CAP36, and CAP156, whose knockdown caused cytokinesis defects similar to CAP50 knockdown, might also disrupt KLIF localization, whereas CAP52 knockdown, which caused similar asymmetrical cytokinesis as CAP46 knockdown, likely has no effect on KLIF localization.

Although the CAP proteins reported in this work appeared to associate with the microtubule cytoskeleton (Fig. S2), it remains unclear whether they bind directly to the cytoskeletal microtubules or indirectly to certain microtubule-associated proteins that bind directly to the cytoskeletal microtubules. It should be noted that none of these CAP proteins contain any known microtubule-binding motifs (Fig. 4*D*), but this does not exclude the possibility that they might contain microtubule-binding motifs that have not been discovered previously, as is the case of the CAPs PAVE1 and PAVE2, which bind directly to microtubules albeit do not contain any known microtubule-binding motifs (26). CAP42 contains a putative amidinotransferase domain (Fig. 3*D*), but whether it has any activity to catalyze the transfer of an amidino group between certain amino acids, such as the transfer of the amidino group from L-Arginine to Glycine catalyzed by the L-Arginine:Glycine amidinotransferase AGAT (E.C. 2.1.4.1), has not been experimentally investigated. However, the localization of CAP42 to the cytoskeleton (Fig. S2) and the requirement of CAP42 for the completion of cytokinesis (Fig. 5) raised an interesting question of whether CAP42 plays a role in modifying the cytoskeletal microtubules through its putative amidinotransferase activity. Nonetheless, future work will be directed to the characterization of these CAPs to determine their biochemical function and potential microtubule-binding activity.

Correct placement of the cleavage furrow (or the cell division plane) is critical for symmetrical cell division and is determined by distinct mechanisms in different organisms (28). Trypanosome cells divide along a preformed cell division fold between the old and the new flagella (4), and the cell division plane appears to be correlated with the length of the new flagellum and/or the new FAZ (29, 30), leading to the proposition that the length of the new flagellum/FAZ determines the cell division plane. Our current finding that depletion of CAP46 and CAP52 impaired the placement of the cleavage furrow (Figs. 6 and 7) and the previous work on TbAIR9’s role in furrow placement (21) suggest that determination of the cell division plane also requires certain CAPs, which may modify the cytoskeletal microtubules to assist the formation of the cell division fold at the correct location. Both CAP46 and CAP52 contain multiple CC motifs (Fig. 3*D*), and they might be parts of a larger protein complex that plays a pivotal role in cleavage furrow placement. Although the CAP46–CAP52 complex and TbAIR9 are both required for furrow positioning, they have distinct localizations on the cytoskeleton. TbAIR9 is distributed throughout the cytoskeleton (21), whereas the CAP46–CAP52 complex is enriched at the anterior and the dorsal edge of the cell (Fig. S2). It is unclear whether TbAIR9 interacts with the CAP46–CAP52 complex, but it would be interesting to investigate the potential functional interplay between TbAIR9 and the CAP46–CAP52 complex.

In summary, we have determined the requirement of the KMD in KLIF for cytokinesis and revealed the role of KLIF in facilitating cleavage furrow ingression by promoting the resolution of the nascent posterior of the OFD cell of a dividing binucleated cell. We further identified a cohort of KLIF-proximal CAPs as essential cytokinesis regulators and demonstrated that these proteins played distinct functions in positioning the cleavage furrow for symmetrical cytokinesis and promoting the ingression of the cleavage furrow for cytokinesis completion.

## Experimental procedures

### Trypanosome cell culture and RNAi

The *T. brucei* strain 29-13 (31) was cultured in SDM-79 medium supplemented with 10% heat-inactivated fetal bovine serum (Atlanta Biologicals, Inc), 15 μg/ml G418, and 50 μg/ml hygromycin B at 27 °C. The *T. brucei* strain SmOx (32) was cultured in SDM-79 medium containing 10% heat-inactivated fetal bovine serum and 1.0 μg/ml puromycin at 27 °C. Cells were diluted with fresh medium whenever the cell density reached 5 × 10^6^ cells/ml.

RNAi was carried out by transfecting the 29-13 strain or the SmOx strain with the pZJM vector containing a DNA fragment of the gene of interest. Specifically, a 548-bp fragment of the KLIF coding sequence (nt. 1143-1690) (12), a 560-bp fragment of the CAP27 coding sequence (nt. 7-566), a 574-bp fragment of the CAP36 coding sequence (nt. 177-750), a 1125-bp fragment of the CAP42 coding sequence (nt. 1-1125), a 1194-bp fragment of the CAP46 coding sequence (nt. 4-1197), a 517-bp fragment of CAP50 coding sequence (nt. 823-1339), a 546-bp fragment of CAP52 coding sequence (nt. 808-1353), and a 585-bp fragment of the CAP156 coding sequence (nt. 1884-2468) were each cloned into the pZJM vector. The resulting plasmids were each linearized by NotI restriction digestion and electroporated into the *T. brucei* 29-13 strain (for CAP proteins) or the SmOx strain (for KLIF). Successful transfectants were selected with 2.5 μg/ml phleomycin, and clonal cell lines were obtained by limiting dilution of the transfectant in a 96-well plate containing SDM-79 medium supplemented with 20% fetal bovine serum and appropriate antibiotics. To induce RNAi, these RNAi cell lines were incubated with 1.0 μg/ml tetracycline, and cell growth was monitored daily.

Two clonal cell lines for each RNAi cell line were selected for analysis, including the plotting of growth curves and the counting of cells with different numbers of kinetoplast and nucleus. Three independent repeats were performed for each clonal cell line, and only the results from one clonal cell line were presented, due to the almost identical phenotypes between the two clonal cell lines.

### Generation of KLIF RNAi complementation cell lines

To generate KLIF RNAi complementation cell lines, a 519-bp fragment of the 3′UTR of KLIF was cloned into the pZJM vector (33). The resulting plasmid was electroporated into the *T. brucei* 29-13 strain. Successful transfectants were selected with 2.5 μg/ml phleomycin, and clonal cell lines (herein designated as KLIF-3′UTR RNAi cell line) were generated by limiting dilution in a 96-well plate. Subsequently, the full-length KLIF gene, the full-length KLIF gene harboring point mutations on the codons encoding two of the most important residues within the nucleotide-binding sites (G230, K231) of the KMD, and the KLIF gene lacking the sequence encoding the C-terminal putative TPM domains were each cloned into the pLew100-3HA-PAC vector (34). The resulting plasmids, pLew100-KLIF-3HA-PAC, pLew100-KLIF^AA^-3HA-PAC, and pLew100-KLIF^KMD^-3HA-PAC, were each transfected into the KLIF-3′UTR RNAi cell line. Successful transfectants were selected with 1.0 μg/ml puromycin and cloned by limiting dilution in a 96-well plate. To induce KLIF-3′UTR RNAi and ectopic expression of KLIF-3HA and its mutants, cells were incubated with 1.0 μg/ml tetracycline, and cell growth was monitored daily.

### *In situ* epitope tagging of proteins

Epitope tagging of proteins from one of their respective endogenous loci was performed by PCR-based epitope tagging method (35). Transfectants were selected with 10 μg/ml blasticidin and cloned by limiting dilution in a 96-well plate. For co-immunoprecipitation of CAP46 and CAP52, CAP46 was endogenously tagged with a C-terminal triple HA epitope in the *T. brucei* 427 cells expressing CAP52-PTP from its endogenous locus. The transfectants were selected with 1.0 μg/ml puromycin and 10 μ/ml blasticidin and cloned by limiting dilution in a 96-well plate.

### Co-immunoprecipitation and Western blotting

Trypanosome cells (∼10^8^) were lysed in 0.5 ml IP buffer (25 mM Tris-HCl, pH 7.4, 100 mM NaCl, 1 mM DTT, 0.07% NP-40, 5% glycerol and protease inhibitor cocktail), and the cell lysate was cleared by centrifugation. For IP of PTP-tagged proteins, the supernatant was incubated with 25 μl settled IgG Sepharose beads (GE Healthcare) for 30 min at 4 °C. Beads were washed five times with the IP buffer, and bound proteins were eluted with 10% SDS. Immunoprecipitated proteins were separated by SDS-PAGE, transferred onto a PVDF membrane, and immunoblotted with anti-HA mAb (clone HA-7, H9658, Sigma-Aldrich, 1:5000 dilution) to detect 3HA-tagged proteins and anti-Protein A polyclonal antibody (anti-ProtA; P3775, Sigma-Aldrich, 1:5000 dilution) to detect PTP-tagged proteins.

### Immunofluorescence microscopy

*T. brucei* cells were adhered to the glass coverslip for 30 min at room temperature, fixed with cold methanol (−20 °C) for 30 min, and rehydrated with PBS for 10 min. Cells were then blocked with 3% BSA in PBS for 30 min at room temperature and incubated with FITC-conjugated anti-HA monoclonal antibody (Clone HA-7, H7411, Sigma-Aldrich, 1:400 dilution) or anti-Protein A polyclonal antibody (Sigma-Aldrich, 1:400 dilution) at room temperature for 60 min. Cells on the coverslip were washed three times with PBS and then incubated with Cy3-conjugated anti-rabbit IgG (C2306, Sigma-Aldrich, 1:400 dilution) at room temperature for 60 min. Cells on the slides were washed three times with PBS and air dried. The slides were mounted in VectaShield mounting medium (Vector Laboratories) containing DAPI and examined using an inverted microscope (model IX71, Olympus) equipped with a cooled CCD camera (model Orca-ER, Hamamatsu) and a PlanApo N 60 × 1.42 NA lens. Images were acquired and processed using Slide-book software (Intelligent Imaging Innovations, Inc).

### Scanning and transmission electron microscopy

Scanning electron microscopic analysis of *T. brucei* cells was carried out using the method described previously (13). Cells were directly fixed with 2.5% (v/v) glutaraldehyde in media for 2 h at room temperature and then settled onto glass coverslips for 30 min after washing three times with PBS. Cells were washed time times with double-distilled water and then dehydrated with a series of alcohol (30%, 50%, 70%, 90%, and 100%) for 5 min each at room temperature. After the cells were dried by critical point drying, the glass coverslips were coated with a 5-nm metal film (Pt:Pd 80:20, Ted Pella Inc) using a sputter coater (Cressington Sputter Coated 208 HR, Ted Pella Inc) and then examined using Nova NanoSEM 230 (FEI). The parameters used were 5 mm for the scanning work distance and 8 kV for the accelerating high voltage.

Preparation of whole-mount cytoskeleton of trypanosome cells was performed according to published procedures (20, 36). Trypanosome cells were harvested by centrifugation, washed twice with PBS, settled onto freshly charged carbon and Formvar-coated grids, and then incubated with PEME buffer containing 1% Nonidet P-40 for 5 min. Cells on the grids were fixed with glutaraldehyde for 20 s, stained with 1% uranyl acetate, and imaged using a JEOL 1400 TEM equipped with a Gatan CCD camera at 60 kV.

### Statistical analysis

Statistical analysis was performed using Chi-square test. Detailed *n* values for each panel in the figures were stated in the corresponding legends. For immunofluorescence microscopy, images were randomly taken and all cells in each image were counted. Data were collected from three independent experiments.

Statistical analysis for the mass spectrometry-identified proteins was performed using the two-tailed unpaired Student’s *t* test in Excel based on the number of unique peptides matched to the same protein identified by mass spectrometry from three biological replicates.

## Data availability

All data are contained within the manuscript.

## Supporting information

This article contains supporting information.

## Conflict of interest

The authors declare that they have no conflicts of interest with the contents of this article.

## References

[1] Gull K. (1999). The cytoskeleton of trypanosomatid parasites. Annu. Rev. Microbiol..

[2] Zhou Q., Hu H., He C.Y., Li Z. (2015). Assembly and maintenance of the flagellum attachment zone filament in *Trypanosoma brucei*. J. Cell Sci..

[3] Sunter J.D., Varga V., Dean S., Gull K. (2015). A dynamic coordination of flagellum and cytoplasmic cytoskeleton assembly specifies cell morphogenesis in trypanosomes. J. Cell Sci..

[4] Wheeler R.J., Scheumann N., Wickstead B., Gull K., Vaughan S. (2013). Cytokinesis in *Trypanosoma brucei* differs between bloodstream and tsetse trypomastigote forms: Implications for microtubule-based morphogenesis and mutant analysis. Mol. Microbiol..

[5] Tu X., Kumar P., Li Z., Wang C.C. (2006). An aurora kinase homologue is involved in regulating both mitosis and cytokinesis in *Trypanosoma brucei*. J. Biol. Chem..

[6] Li Z., Wang C.C. (2006). Changing roles of aurora-B kinase in two life cycle stages of *Trypanosoma brucei*. Eukaryot. Cell.

[7] Kumar P., Wang C.C. (2006). Dissociation of cytokinesis initiation from mitotic control in a eukaryote. Eukaryot. Cell.

[8] Hammarton T.C., Kramer S., Tetley L., Boshart M., Mottram J.C. (2007). *Trypanosoma brucei* Polo-like kinase is essential for basal body duplication, kDNA segregation and cytokinesis. Mol. Microbiol..

[9] Li Z., Lee J.H., Chu F., Burlingame A.L., Gunzl A., Wang C.C. (2008). Identification of a novel chromosomal passenger complex and its unique localization during cytokinesis in *Trypanosoma brucei*. PLoS One.

[10] Li Z., Umeyama T., Wang C.C. (2009). The aurora kinase in *Trypanosoma brucei* plays distinctive roles in metaphase-anaphase transition and cytokinetic initiation. PLoS Pathog..

[11] McAllaster M.R., Ikeda K.N., Lozano-Nunez A., Anrather D., Unterwurzacher V., Gossenreiter T., Perry J.A., Crickley R., Mercadante C.J., Vaughan S., de Graffenried C.L. (2015). Proteomic identification of novel cytoskeletal proteins associated with TbPLK, an essential regulator of cell morphogenesis in *Trypanosoma brucei*. Mol. Biol. Cell.

[12] Zhou Q., An T., Pham K.T.M., Hu H., Li Z. (2018). The CIF1 protein is a master orchestrator of trypanosome cytokinesis that recruits several cytokinesis regulators to the cytokinesis initiation site. J. Biol. Chem..

[13] Zhou Q., Gu J., Lun Z.R., Ayala F.J., Li Z. (2016). Two distinct cytokinesis pathways drive trypanosome cell division initiation from opposite cell ends. Proc. Natl. Acad. Sci. U. S. A..

[14] Zhou Q., Hu H., Li Z. (2016). An EF-hand-containing protein in *Trypanosoma brucei* regulates cytokinesis initiation by maintaining the stability of the cytokinesis initiation factor CIF1. J. Biol. Chem..

[15] Kurasawa Y., Hu H., Zhou Q., Li Z. (2018). The trypanosome-specific protein CIF3 cooperates with the CIF1 protein to promote cytokinesis in *Trypanosoma brucei*. J. Biol. Chem..

[16] Hilton N.A., Sladewski T.E., Perry J.A., Pataki Z., Sinclair-Davis A.N., Muniz R.S., Tran H.L., Wurster J.I., Seo J., de Graffenried C.L. (2018). Identification of TOEFAZ1-interacting proteins reveals key regulators of *Trypanosoma brucei* cytokinesis. Mol. Microbiol..

[17] Hu H., An T., Kurasawa Y., Zhou Q., Li Z. (2019). The trypanosome-specific proteins FPRC and CIF4 regulate cytokinesis initiation by recruiting CIF1 to the cytokinesis initiation site. J. Biol. Chem..

[18] Pham K.T.M., Zhou Q., Kurasawa Y., Li Z. (2019). BOH1 cooperates with Polo-like kinase to regulate flagellum inheritance and cytokinesis initiation in *Trypanosoma brucei*. J. Cell Sci..

[19] Zhang X., An T., Pham K.T.M., Lun Z.R., Li Z. (2019). Functional analyses of cytokinesis regulators in bloodstream stage *Trypanosoma brucei* parasites identify functions and regulations specific to the life cycle stage. mSphere.

[20] Sherwin T., Gull K. (1989). Visualization of detyrosination along single microtubules reveals novel mechanisms of assembly during cytoskeletal duplication in trypanosomes. Cell.

[21] May S.F., Peacock L., Almeida Costa C.I., Gibson W.C., Tetley L., Robinson D.R., Hammarton T.C. (2012). The *Trypanosoma brucei* AIR9-like protein is cytoskeleton-associated and is required for nucleus positioning and accurate cleavage furrow placement. Mol. Microbiol..

[22] Schock M., Schmidt S., Ersfeld K. (2021). Novel cytoskeleton-associated proteins in trypanosoma brucei are essential for cell morphogenesis and cytokinesis. Microorganisms.

[23] Brasseur A., Rotureau B., Vermeersch M., Blisnick T., Salmon D., Bastin P., Pays E., Vanhamme L., Perez-Morga D. (2013). Trypanosoma brucei FKBP12 differentially controls motility and cytokinesis in procyclic and bloodstream forms. Eukaryot. Cell.

[24] Olego-Fernandez S., Vaughan S., Shaw M.K., Gull K., Ginger M.L. (2009). Cell morphogenesis of Trypanosoma brucei requires the paralogous, differentially expressed calpain-related proteins CAP5.5 and CAP5.5V. Protist.

[25] Dean S., Sunter J.D., Wheeler R.J. (2017). TrypTag.org: A trypanosome Genome-wide protein localisation resource. Trends Parasitol..

[26] Sinclair A.N., Huynh C.T., Sladewski T.E., Zuromski J.L., Ruiz A.E., de Graffenried C.L. (2021). The Trypanosoma brucei subpellicular microtubule array is organized into functionally discrete subdomains defined by microtubule associated proteins. PLoS Pathog..

[27] Balasubramanian M.K., Helfman D.M., Hemmingsen S.M. (1992). A new tropomyosin essential for cytokinesis in the fission yeast *S. pombe*. Nature.

[28] Oliferenko S., Chew T.G., Balasubramanian M.K. (2009). Positioning cytokinesis. Genes Dev..

[29] Kohl L., Robinson D., Bastin P. (2003). Novel roles for the flagellum in cell morphogenesis and cytokinesis of trypanosomes. EMBO J..

[30] Zhou Q., Liu B., Sun Y., He C.Y. (2011). A coiled-coil- and C2-domain-containing protein is required for FAZ assembly and cell morphology in *Trypanosoma brucei*. J. Cell Sci..

[31] Wirtz E., Leal S., Ochatt C., Cross G.A. (1999). A tightly regulated inducible expression system for conditional gene knock-outs and dominant-negative genetics in *Trypanosoma brucei*. Mol. Biochem. Parasitol..

[32] Poon S.K., Peacock L., Gibson W., Gull K., Kelly S. (2012). A modular and optimized single marker system for generating Trypanosoma brucei cell lines expressing T7 RNA polymerase and the tetracycline repressor. Open Biol..

[33] Wang Z., Morris J.C., Drew M.E., Englund P.T. (2000). Inhibition of *Trypanosoma brucei* gene expression by RNA interference using an integratable vector with opposing T7 promoters. J. Biol. Chem..

[34] Wei Y., Hu H., Lun Z.R., Li Z. (2014). Centrin3 in trypanosomes maintains the stability of a flagellar inner-arm dynein for cell motility. Nat. Commun..

[35] Shen S., Arhin G.K., Ullu E., Tschudi C. (2001). *In vivo* epitope tagging of *Trypanosoma brucei* genes using a one step PCR-based strategy. Mol. Biochem. Parasitol..

[36] Hu H., Hu L., Yu Z., Chasse A.E., Chu F., Li Z. (2012). An orphan kinesin in trypanosomes cooperates with a kinetoplastid-specific kinesin to maintain cell morphology by regulating subpellicular microtubules. J. Cell Sci..

