# Supplementary figures and images for: KLIF-associated cytoskeletal proteins in Trypanosoma brucei regulate cytokinesis by promoting cleavage furrow positioning and ingression
Source: J Biol Chem. 2022 Apr 18;298(6):101943. doi: 10.1016/j.jbc.2022.101943 (PMC9117871; doi:10.1016/j.jbc.2022.101943)

Figure S1

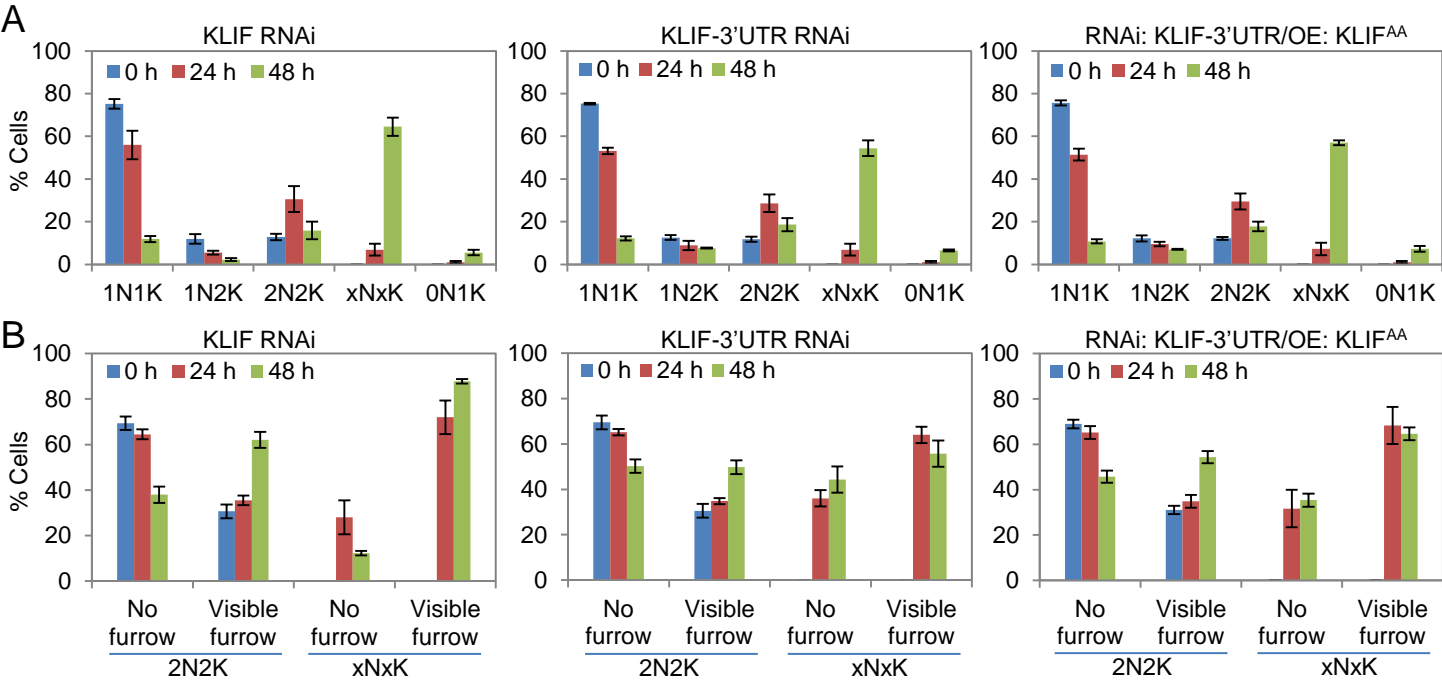

Supplement: Supplemental Figure S1 [file mmc2.pdf]

Figure S2

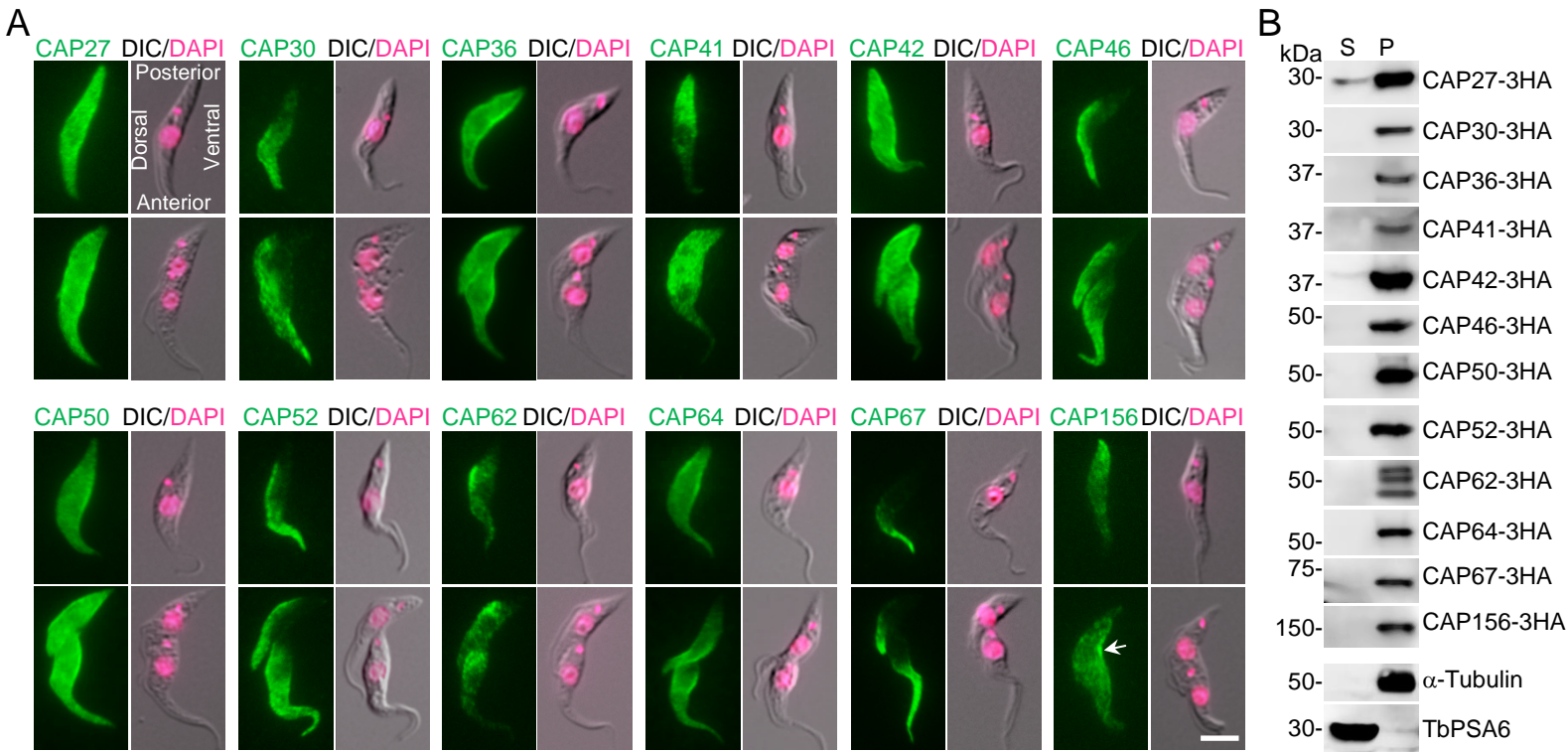

Supplement: Supplemental Figure S2 [file mmc3.pdf]
